# Enhanced collaboration of physicians and speech-language therapists promotes early oral intake in older adults with aspiration pneumonia: A retrospective cohort study

**DOI:** 10.1371/journal.pone.0350495

**Published:** 2026-06-10

**Authors:** Akinori Tawaraya, Atsushi Keyaki

**Affiliations:** Center for Rehabilitation, Takatsuki General Hospital, Takatsuki City, Osaka, Japan; Public Library of Science, UNITED KINGDOM OF GREAT BRITAIN AND NORTHERN IRELAND

## Abstract

**Background & aims:**

Early swallowing assessment and initiation of oral intake are important in the management of aspiration pneumonia in older adults. However, the factors influencing early initiation of oral intake have not been widely studied. We implemented a system of enhanced collaboration between physicians and speech-language therapists (ECPS) as a structured inter-professional clinical pathway to standardize decision-making and facilitate early oral intake. We hypothesized that ECPS would increase the rate of early oral intake within two days of admission.

**Methods:**

This retrospective cohort study included inpatients aged ≥65 years with aspiration pneumonia (ECPS and control groups). Multivariate logistic regression was used to evaluate the associations of ECPS with early oral intake. Secondary outcomes assessed safety in an exploratory analysis, examining whether early oral intake was associated with disadvantages such as longer duration of antibiotic therapy, recurrence of aspiration pneumonia, decline in swallowing function, need for artificial nutrition, change in residence, or longer hospital stay.

**Results:**

The ECPS group comprised 160 of the 300 eligible patients. ECPS was associated with increased odds of early oral intake success (OR = 3.85, 95% CI: 2.22–6.70, *p* < 0.001). Furthermore, delayed swallowing assessment was associated with decreased odds of early oral intake success (OR = 0.69, 95% CI: 0.53–0.90, *p* < 0.01). In exploratory analysis, early oral intake was not associated with any clear increase in disadvantages.

**Conclusion:**

Structured collaboration between physicians and swallowing specialists regarding early oral intake could represent a factor worth considering in achieving effective management in older adults with aspiration pneumonia.

## Introduction

Aspiration pneumonia (AP) is a pulmonary infection that occurs after the inhalation of oropharyngeal secretions, food, liquids, or gastric contents into the lower respiratory tract. There are community-acquired and healthcare-associated forms, and AP often occurs in people with dysphagia or aspiration into the lungs [1]*.* Diagnostic criteria typically include clinical symptoms (e.g., cough, fever, dyspnea), radiographic evidence indicative of aspiration, and the presence of risk factors such as dysphagia, altered mental status, or gastroesophageal reflux [2–4]. AP predominantly affects older adults, and it poses significant risks for recurrent pneumonia and increased mortality [5,6]. Optimizing early management of AP is therefore thought to be important [7]. Current best-practice management strategies for AP typically encompass the judicious selection of antibiotics, comprehensive supportive care (such as oxygen therapy and the management of fluids and nutrition), and the implementation of preventive measures through inter-professional collaboration, including swallowing assessment, rehabilitation, and positioning strategies [1,4,8,9]. Within this framework, early swallowing assessment and the initiation of oral intake are considered to be central components of management [8–12]. However, nil per os policies are often customary during the initial management of AP [13]. Importantly, the factors influencing early initiation of oral intake have not been widely studied, and reports on the implementation of early oral intake practices in patients with AP are insufficient [14–18]. An evidence–practice gap therefore exists regarding the management of AP, particularly in the early initiation of oral intake [19,20].

To give context, under Japanese law, speech language therapists (SLTs) provide dysphagia-related care under the supervision of physicians, and both swallowing assessment and initiation of oral intake require a physician’s authorization. Decision-making from hospital admission to the initiation of oral intake in patients with AP has often therefore relied on the discretion of individual physicians, which theoretically results in delayed initiation of oral intake. We suggest that this gap presents the need for a standardized inter-professional clinical pathway with the aim of reducing variability in decision-making and ensuring timely initiation of oral intake.

At our hospital, patients with AP have been managed since 2017 according to a standard protocol [21]. This protocol includes initial diagnostic tests, infection control, oxygen and antibiotic management, early rehabilitation and swallowing assessment, diet modification, caregiver education, and preventive measures such as vaccination and advance care planning. Enhanced collaboration between physicians and speech-language therapists (ECPS) was implemented as part of this protocol as a structured inter-professional clinical pathway aiming to standardize decision-making and streamline the process from hospital admission to initiation of oral intake in patients with AP. ECPS incorporates an inter-professional agreement, a standardized management protocol, and a coordinated clinical workflow.

We hypothesized that implementation of ECPS would facilitate earlier initiation of oral intake in patients with AP. In this study, we evaluate the association between implementation of ECPS and the timing of oral intake initiation, thereby examining the effectiveness of a standardized clinical workflow in routine practice.

## Methods

### Study design and setting

This retrospective cohort study was conducted at the Center for Rehabilitation, Takatsuki General Hospital, Osaka, Japan. The hospital functions as a general medical center for approximately 340,000 residents. The cohort consisted of consecutive patients hospitalized with AP for at least one day during the inclusion period (April 1, 2015-March 31, 2019), as identified from the Japanese Diagnosis Procedure Combination system. For patients who were admitted to the hospital more than once, we used data from their initial admission only. The study protocol defined the study conduct period as October 2019 to March 2022. The authors had access to identifiable information during data extraction, but all data were anonymized prior to analysis.

### Participants

Included in this study were patients aged ≥65 years and newly admitted to our hospital with AP. They came from various settings, including private homes, nursing homes, and other hospitals. AP was diagnosed by physicians in accordance with published guidelines at the time of admission [2]. In addition to radiological evidence of pneumonia and characteristic clinical history (e.g., dysphagia and a known or strongly suspected episode of aspiration), the presence of risk factors for aspiration were required for diagnosis. Pneumonia was confirmed by the presence of a new gravity-dependent infiltrate on chest radiography or computed tomography that was accompanied by clinical features including fever, elevated white blood cell count or C-reactive protein level, and respiratory symptoms, such as sputum production [1–3]. Patients diagnosed with AP were primarily managed by the Department of Internal Medicine. All participants in this study underwent dysphagia rehabilitation based on the system used in our hospital.

We excluded patients based on pre-established criteria: those who died during hospitalization, those who were not prescribed physical therapy or speech language therapy, those who had already ceased oral intake prior to the onset of the disease, those with AP due to vomiting (as this condition is more consistent with acute chemical aspiration than dysphagia-related aspiration), those who never received oral intake due to poor general condition after admission, those who did not undergo antibiotic therapy, those who developed cerebrovascular or gastrointestinal complications during their post-admission period, and those with missing or incomplete data.

### Our dysphagia rehabilitation system

All patients with AP received dysphagia rehabilitation within this system. A swallowing assessment was performed by an SLT in accordance with published guidelines [22]. The assessment included cognitive and respiratory function, cervical range of motion, oral function, voice and articulation, dehydration and nutritional status, swallowing screening (repetitive saliva swallowing test, modified water swallowing test, and food test), and instrumental examinations when indicated.

Eligibility for oral intake was determined according to predefined institutional criteria developed by SLTs, based on standardized swallowing screening results and comprehensive clinical assessment. The dysphagia diet was one of five levels [23], with the appropriate diet level being selected according to the patient’s swallowing function and overall clinical status. If oral intake difficulty was identified, ongoing dysphagia rehabilitation and reassessment were conducted over the subsequent 1–2 weeks. Instrumental evaluations, including fiber optic endoscopic evaluation of swallowing and videofluoroscopic swallowing studies, were performed according to the patient’s condition. SLTs provided continuous rehabilitation and adjusted the eating and swallowing environment (dietary texture, posture, and related factors) while considering post-discharge feasibility. Swallowing function was promptly reassessed if clinical deterioration occurred. SLTs were also involved in coordinating oral care, respiratory rehabilitation, nutritional management, discharge planning, and inter-facility communication within the multidisciplinary team. Multidisciplinary collaboration ensured individualized goal setting and continued support until discharge.

### Exposure

The exposure of interest is ECPS, which was introduced in April 2017 for patients with AP. During the inclusion period (April 1, 2015-March 31, 2019), patients admitted before March 2017 were assigned to the control group, and those admitted from April 2017 onward were assigned to the ECPS group. This symmetrical observation window was selected to balance potential secular trends over time. The end date was set prior to the onset of the COVID-19 pandemic in Japan to avoid confounding related to pandemic-associated changes.

The intervention consisted of three components:

1. **Inter-professional agreement**:
  The inter-professional agreement underlying ECPS was established through interdisciplinary discussions and consensus among physicians and SLTs within the institution prior to implementation. This agreement was based on a shared recognition, informed by previous studies [1,9,16,20,30], of the importance of early swallowing assessment and timely initiation of oral intake in patients with AP.
2. **Standardized management protocol** [21]:
  The inter-professional agreement was directly incorporated into the institutional AP treatment protocol and immediately implemented into routine clinical practice. The protocol specifies that physicians oversee SLT-led assessment of oral hygiene and swallowing function within 1–2 days of admission in clinically stable patients. Based on the absence of high-risk conditions (e.g., altered mental status, respiratory distress/failure, or hemodynamic instability), physicians determined whether oral intake could be initiated and entered diet orders when clinically appropriate.
3. **Coordinated clinical workflow**:
  Physicians ordered a swallowing assessment from a SLT upon admission and entered diet orders in advance if deemed clinically appropriate. To avoid delays, SLTs tried to respond promptly to consultation requests. When a diet order had already been entered, swallowing assessment and initiation of oral intake were performed at the initial assessment. In principle, the first diet consisted of jelly and pureed food unless there were special circumstances.

**Fig 1:**
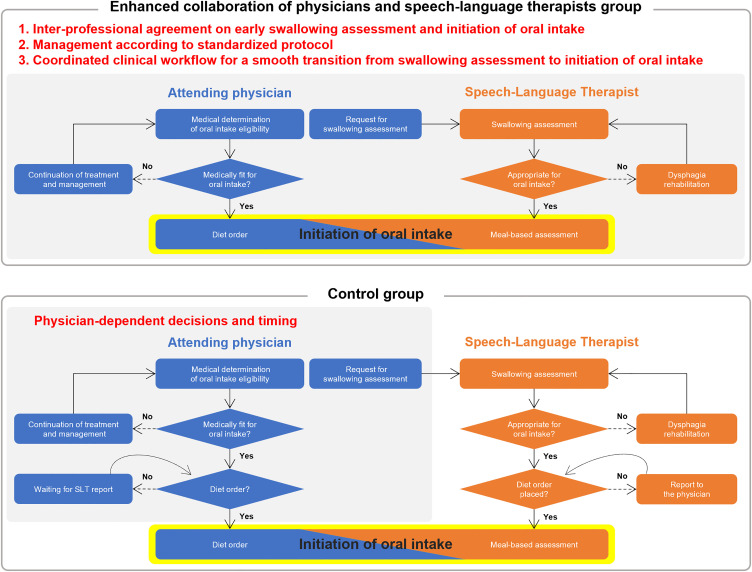
Exposure: Overview of the process from swallowing assessment to oral intake (schematic). Note: This figure provides a simplified overview of the process for illustrative purposes. Not all clinical details or exceptions are shown.

In contrast, in the control group from the earlier period, decisions regarding swallowing assessment and initiation of oral intake were made at the discretion of individual attending physicians, and without a predefined inter-professional agreement, standardized protocol, or coordinated workflow. The workflow is illustrated in Fig 1.

Within the ECPS system, we reorganized and standardized the clinical workflow and timing of decisions. However, during the inclusion period there were no changes to the formal decision-making authority, the clinical criteria used by SLTs to assess eligibility for oral intake, or to the content of dysphagia rehabilitation.

### Baseline covariates

Baseline characteristics of patients at the time of admission were: age, sex, pre-admission residence, long-term care insurance system, aspiration risk conditions, ‘A-DROP’ (age, dehydration, respiratory failure, orientation disturbance, low blood pressure) [2,24], geriatric nutritional risk index (GNRI) [25], functional independence measure (FIM), and functional oral intake scale (FOIS) [26] before onset.

Aspiration risk conditions included stroke, neuromuscular diseases and dementia, as specified in the guidelines [1–3,27]. In addition, a history of AP was also included as an aspiration risk condition because it was considered to be a potential confounding factor. A-DROP is a validated severity classification of pneumonia that was developed for Japanese people based on CURB-65 [28]. A higher A-DROP score indicates severe pneumonia. The FIM is an assessment measure of activities of daily living (ADL) that consists of 13 motor and five cognitive items, each rated on a 7-point scale. Scores range from 18 to 126, with higher scores indicating better functional independence. The FOIS is a measure of swallowing function consisting of seven levels, ranging from level 1 (nothing by mouth) to level 7 (total oral intake with no restrictions). The FOIS score before onset was determined retrospectively based on information regarding dietary status prior to illness onset, obtained from nursing summaries, referral letters, and admission interview records. Diet form was coded according to the Japanese Dysphagia Diet 2021 established by the Japanese Society of Dysphagia Rehabilitation [23], using a predefined mapping rule. The presence or absence of alternative nutrition was also considered when assigning the FOIS level. FOIS scoring was performed retrospectively by one of the authors (AT), who was trained in swallowing assessment.

### Primary outcome

The primary outcome of this study was early oral intake within 2 days. Early oral intake was operationally defined as the initiation of oral intake within two calendar days after admission, counting the day of admission as day 0. Patients who initiated oral intake within this period (days 0–2) were classified into the ‘oral intake within two days’ group.

Oral intake was defined as the consumption of any nutritional substance, including caloric jelly, but not thickened water alone. This definition does not take into account either the caloric content or the volume per diet. The presence or absence of oral intake was confirmed by reviewing progress notes in the electronic medical records, as entered by nursing staff.

### Secondary outcomes

Secondary outcomes were safety endpoints. This exploratory analysis aimed to confirm whether early oral intake was associated with increased disadvantages. To this end, we compared disadvantages (i.e., duration of antibiotic therapy, resumption of antibiotics due to recurrence of AP, change of residence, decline in swallowing function, need for artificial nutrition at discharge, and length of hospital stay) between patients who did and did not initiate oral intake within two days. Changes in the FOIS score were assessed by comparing the scores before onset and at discharge. A decrease of even one point was considered to be indicative of a decline in FOIS. The discharge FOIS score was determined by an SLT at the time of discharge. The classification of artificial nutrition at discharge is based on the FOIS score, with ≤ level 3 indicating the presence of artificial nutrition.

### Post-admission clinical outcomes

Post-admission process variables were: time to SLT assessment, time to oral intake initiation. Clinical outcomes after initiation of management were: early oral intake (within 2 days), duration of antibiotic therapy, resumption of antibiotic therapy, length of hospital stay, FIM at discharge, FIM gain, post-discharge residence, change of residence, FOIS at discharge, decline in FOIS, and artificial nutrition at discharge.

FIM gain is the difference in FIM between admission and discharge and represents the change in ADL. The FIM score was scored by a physical therapist.

### Study size

The sample size was estimated based on the requirements for logistic regression analysis. At least 10 events per predictor variable are generally considered necessary for stable regression estimates [29]; we therefore conservatively assumed 15 events per predictor variable. Assuming the inclusion of 10 independent variables in the model, at least 150 events were required. The primary outcome of this study was successful oral intake within two days. Based on previous reports that indicated early oral intake success rates of 54.3% to 66.1% in patients with AP [14–16,18,30], the expected event rate was approximately 60%. The total sample size required to achieve the target number of events was therefore estimated to be about 250 patients. To account for potential missing or incomplete data, we added an additional 10%, resulting in a final target sample size of approximately 275 patients.

### Statistical analysis

All quantitative variables were treated as continuous. The normality of these variables was assessed using the Shapiro-Wilk test. Results are presented as mean (SD) for normally distributed variables and as median (IQR) for non-normally distributed variables. Additionally, all categorical variables were dummy-coded. For univariate analyses, normally distributed continuous variables were analyzed using t-tests, whereas non-normally distributed variables and categorical variables were analyzed using the χ² test, Fisher’s exact test, or the Mann-Whitney U test, as appropriate. Effect sizes are reported as r for t-tests and non-parametric tests, and as Cramer’s V for the χ² test and Fisher’s exact test. Values of 0.10–0.30 were considered small, 0.30–0.50 moderate, and >0.50 large; values <0.10 were considered negligible [31].

The primary outcome was assessed by comparing the ECPS group and the control group. Multivariate logistic regression analysis was conducted to investigate whether ECPS is an independent factor associated with oral intake within two days. Explanatory variables included age; sex (reference: male); pre-admission residence (reference: home); use of the long-term care insurance system (reference: non-use); presence of aspiration risk conditions (reference: absent); A-DROP; GNRI; total FIM at admission; FOIS before onset; Time to SLT assessment; and ECPS. Explanatory variables were selected based on clinical relevance and temporal sequence. Baseline patient characteristics at admission were included as potential confounders. ECPS and time to SLT assessment were included as clinically relevant process-related variables occurring after admission. All explanatory variables were entered simultaneously into the model using the forced-entry method.

An exploratory analysis of safety endpoints was conducted as a secondary outcome. Within the ECPS and control groups, we compared patients who initiated oral intake within two days with those who did not. Statistical analyses were conducted using EZR ver. 1.55 (Easy R) [32]. A value of *p* < 0.05 was employed as the threshold for statistical significance.

### Ethics

This study was approved by the Takatsuki General Hospital Ethics Committee (Approval No. 2019-55), and it was conducted in accordance with the Japanese Ethical Guidelines for Medical and Health Research Involving Human Subjects. Consent to inclusion in the study was obtained from the patients via an opt-out process on the hospital website.

## Results

### Baseline patient characteristics

**Fig 2:**
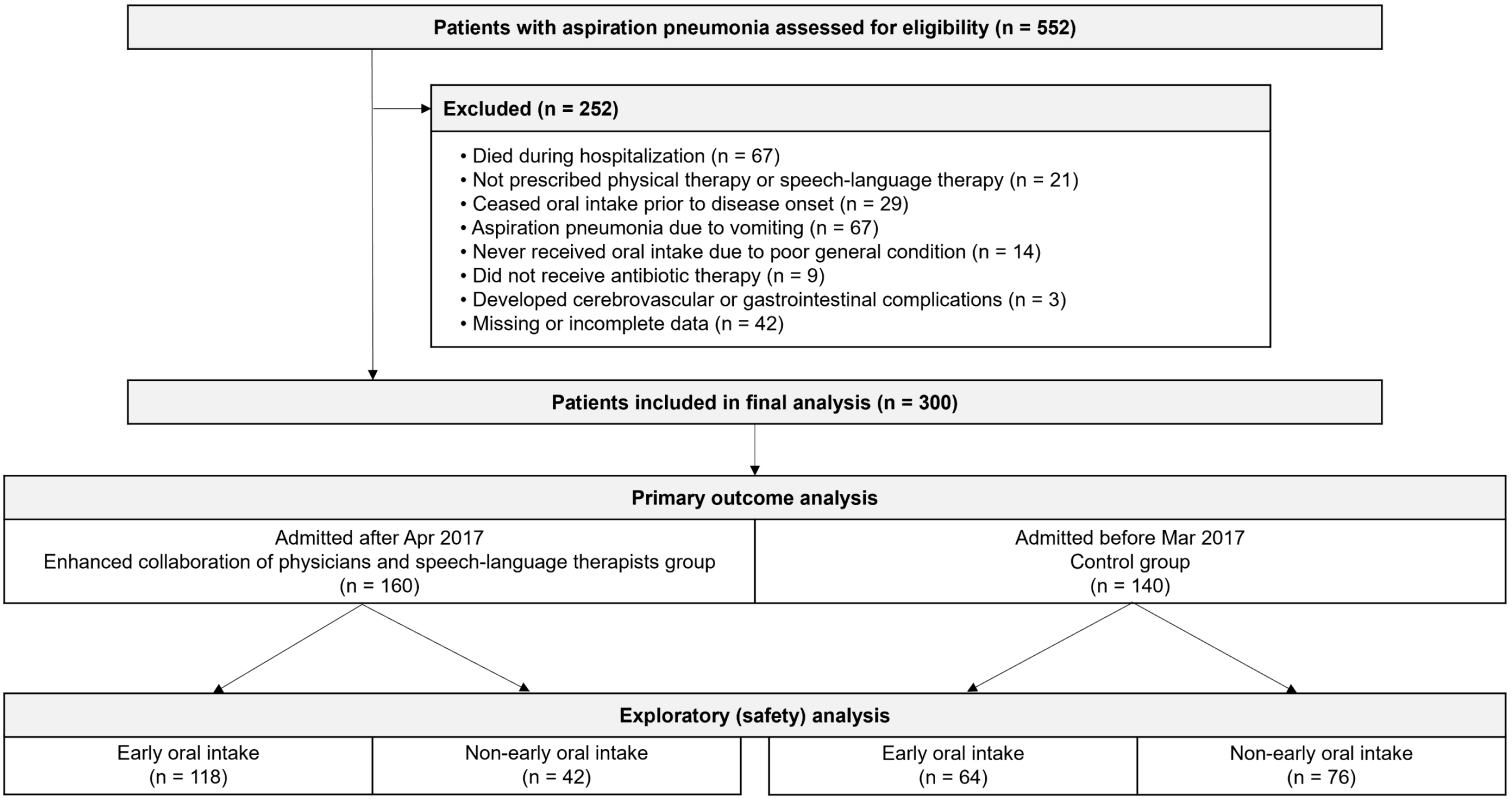
Flow diagram of patient selection. Note: The system of enhanced collaboration of physicians and speech-language therapists was introduced in April 2017; groups were defined by admission date.

Initially, data were collected from 552 patients with AP who met the inclusion criteria. Of these, we excluded 252 patients based on pre-established criteria (Fig 2). Data from 300 patients were therefore examined.

Baseline characteristics of patients are shown in Table 1. The 300 patients examined had a median (IQR) age of 87 (81–90) years. The ECPS group comprised 160 participants. A large proportion of patients displayed the following characteristics: residence outside the home, utilization of long-term care insurance, presence of aspiration risk conditions, moderate-to-severe pneumonia, nutrition-related risks, and low ADLs. More than half of the patients had a FOIS score of ≤6, and swallowing dysfunction was common among them. No statistically significant differences were observed between the ECPS and control groups, and the effect sizes for all variables were below 0.10.

**Table 1:** Baseline patient characteristics.

	All n = 300	ECPS n = 160	Control n = 140	*p* value	effect size
Age (years), median (IQR)	87 (81-90)	87 (81-91)	86 (81-90)	0.387	r = 0.05
Sex, n (%)					
Male, n (%)	165 (55.0)	83 (51.9)	82 (58.6)	0.295	V = 0.07
Female, n (%)	135 (45.0)	77 (48.1)	58 (41.4)		
Pre-admission residence, n (%)					
Home, n (%)	150 (50.0)	79 (49.4)	71 (50.7)	0.626	V = 0.06
Nursing home, n (%)	142 (47.3)	78 (48.8)	64 (45.7)		
Hospital, n (%)	8 (2.7)	3 (1.9)	5 (3.6)		
Long-term care insurance system, n (%)					
Support need, n (%)	30 (10.0)	13 (8.1)	17 (12.1)	0.410	V = 0.08
Long-term care, n (%)	247 (82.3)	136 (85.0)	111 (79.3)		
Aspiration risk conditions, n (%)	225 (75.0)	125 (78.1)	100 (71.4)	0.229	V = 0.08
Stroke, n (%)	122 (40.7)	70 (43.8)	52 (37.1)	0.296	V = 0.07
Neuromuscular disease, n (%)	38 (12.7)	22 (13.8)	16 (11.4)	0.668	V = 0.04
Dementia, n (%)	132 (44.0)	74 (46.2)	58 (41.4)	0.470	V = 0.05
History of AP, n (%)	32 (10.7)	14 (8.8)	18 (12.9)	0.336	V = 0.07
A-DROP, median (IQR)	2 (2–3)	2 (2–3)	2 (2–3)	0.772	r = 0.02
0 (Mild), n (%)	2 (0.7)	1 (0.6)	1 (0.7)		
1 (Moderate), n (%)	56 (18.7)	31 (19.4)	25 (17.9)		
2 (Moderate), n (%)	105 (35.0)	54 (33.8)	51 (36.4)		
3 (Severe), n (%)	90 (30.0)	46 (28.7)	44 (31.4)		
4 (Very severe), n (%)	42 (14.0)	25 (15.6)	17 (12.1)		
5 (Very severe), n (%)	5 (1.7)	3 (1.9)	2 (1.4)		
GNRI, mean ± SD	86.5 (11.3)	87.0 (11.2)	85.9 (11.4)	0.396	r = 0.05
No risk, n (%)	54 (18.0)	30 (18.8)	24 (17.1)		
Low risk, n (%)	44 (14.7)	26 (16.2)	18 (12.9)		
Moderate risk, n (%)	94 (31.3)	51 (31.9)	43 (30.7)		
Major risk, n (%)	108 (36.0)	53 (33.1)	55 (39.3)		
Total FIM at admission, median (IQR)	27 (20-41)	27 (19-41)	27 (21-41)	0.588	r = 0.03
Motor FIM, median (IQR)	14 (13–25)	14 (13–26)	14 (13–24)	0.401	r = 0.05
Cognitive FIM, median (IQR)	11 (7–18)	11 (5–17)	12 (8–18)	0.225	r = 0.07
FOIS before onset, median (IQR)	6 (56–7)	6 (56–7)	6 (56–7)	0.790	r = 0.02
Level 4, n (%)	65 (21.7)	36 (22.5)	29 (20.7)		
Level 5, n (%)	19 (6.3)	11 (6.9)	8 (5.7)		
Level 6, n (%)	73 (24.3)	37 (23.1)	36 (25.7)		
Level 7, n (%)	143 (47.7)	76 (47.5)	67 (47.9)		

Data are presented as mean (SD), median (IQR), frequency (%).

Abbreviations: ECPS, enhanced collaboration of physicians and speech-language therapists; AP, aspiration pneumonia; A-DROP, age, dehydration, respiratory failure, orientation disturbance, low blood pressure; GNRI, Geriatric Nutritional Risk Index; FIM, Functional Independence Measure; FOIS, Functional Oral Intake Scale

### Clinical outcomes after initiation of management

The clinical outcomes are shown in Table 2. The median time to SLT assessment was 1 day (IQR: 1–2 days), and the median time to oral intake initiation was 2 days (IQR: 1–4 days). Early oral intake (within 2 days) was achieved in 182 cases (60.7%). The median duration of antibiotic therapy was 6 days (IQR: 5–8 days), and antibiotic treatment was resumed in 32 patients (10.7%). The median length of hospital stay was 15 days (IQR: 11–27 days). At the time of discharge, the median total FIM score was 32 (IQR: 21–54), with a median FIM gain of 0 (IQR: 0–8) for motor functions and 0 (IQR: 0–0) for cognitive functions, indicating minimal change from the initial assessment. Following discharge, there was a drop in the proportion of patients residing in community or institutional settings.

**Table 2:** Clinical outcomes after initiation of management.

	All n = 300	ECPS n = 160	Control n = 140	*p* value	effect size
**Post-admission process variables**					
Time to SLT assessment, median (IQR)	1 (1–2)	1 (1–2)	1 (1–2)	0.023	r = 0.13
Time to oral intake initiation, median (IQR)	2 (1–4)	1 (1–3)	3 (1–4)	< 0.001	r = 0.29
**Clinical outcomes**					
Early oral intake (within 2 days), n (%)	182 (60.7)	118 (73.8)	64 (45.7)	< 0.001	V = 0.29
Duration of antibiotic therapy, median (IQR)	6 (5–8)	5 (4–7)	8 (6–11)	< 0.001	r = 0.40
Resumption of antibiotic therapy, n (%)	32 (10.7)	9 (5.6)	23 (16.4)	0.005	V = 0.18
Length of hospital stay, median (IQR)	15 (11–27)	13 (9–24)	18 (12-31)	< 0.001	r = 0.22
Total FIM at discharge, median (IQR)	32 (21-54)	30 (21-50)	35 (22-55)	0.251	r = 0.07
Motor FIM, median (IQR)	18 (13-37)	17 (13-36)	19 (13-37)	0.516	r = 0.04
Cognitive FIM, median (IQR)	13 (7–18)	13 (6–18)	13 (8–18)	0.220	r = 0.07
Motor FIM gain, median (IQR)	0 (0-8)	0 (0-8)	1 (0-9)	0.156	r = 0.08
Cognitive FIM gain, median (IQR)	0 (0−0)	0 (0−0)	0 (0−0)	0.613	r = 0.03
Post-discharge residence, n (%)					
Home, n (%)	99 (33.0)	53 (33.1)	46 (32.9)	0.998	V = 0.00
Nursing home, n (%)	120 (40.0)	64 (40.0)	56 (40.0)		
Hospital, n (%)	81 (27.0)	43 (26.9)	38 (27.1)		
Change of residence, n (%)	97 (32.3)	53 (33.1)	44 (31.4)	0.850	V = 0.02
FOIS at discharge, median (IQR)	5 (4–6)	5 (4–6)	5 (4–6)	0.318	r = 0.06
Level 1, n (%)	24 (8.0)	8 (5.0)	16 (11.4)		
Level 2, n (%)	20 (6.7)	10 (6.2)	10 (7.1)		
Level 3, n (%)	2 (0.7)	2 (1.2)	0 (0.0)		
Level 4, n (%)	80 (26.7)	47 (29.4)	33 (23.6)		
Level 5, n (%)	94 (31.3)	48 (30.0)	46 (32.9)		
Level 6, n (%)	46 (15.3)	22 (13.8)	24 (17.1)		
Level 7, n (%)	34 (11.3)	23 (14.4)	11 (7.9)		
Decline in FOIS, n (%)	205 (68.3)	100 (62.5)	105 (75.0)	0.028	V = 0.13
Artificial nutrition at discharge, n (%)	46 (15.3)	20 (12.5)	26 (18.6)	0.195	V = 0.08

Data are presented as median (IQR) or frequency (%). Variables in this table were recorded after initiation of management and represent the clinical course and outcomes during hospitalization and at discharge.

Abbreviations: ECPS, enhanced collaboration of physicians and speech-language therapists; SLT, Speech-Language Therapist; FIM, Functional Independence Measure; FOIS, Functional Oral Intake Scale

Compared with the control group, the ECPS group showed significantly shorter times to SLT assessment (1 day, IQR: 1–2 vs. 1 day, IQR: 1–2; *p* = 0.023, r = 0.13) and to oral intake initiation (1 day, IQR: 1–3 vs. 3 days, IQR: 1–4; *p* < 0.001, r = 0.29). Early oral intake (within 2 days) achievement was more common in the ECPS group (73.8% vs. 45.7%; *p* < 0.001, V = 0.29). Antibiotic duration was shorter (5 days, IQR: 4–7 vs. 8 days, IQR: 6–11; *p* < 0.001, r = 0.40), and resumption rates were lower (5.6% vs. 16.4%; *p* = 0.005, V = 0.18). Hospital stays were shorter in the ECPS group (13 days, IQR: 9–24 vs. 18 days, IQR: 12–31; *p* < 0.001, r = 0.22). Decline in FOIS was also less common (62.5% vs. 75.0%; *p* = 0.028, V = 0.13).

### Comparison of baseline characteristics between patients with and without early oral intake (within 2 days)

The results of comparison of baseline characteristics between patients with and without early oral intake are shown in Table 3.

**Table 3:** Comparison of baseline characteristics between patients with and without early oral intake (within 2 days).

	ECPS group				Control group			
	early oral intake n = 118	non-early oral intake n = 42	*p* value	effect size	early oral intake n = 64	non-early oral intake n = 76	*p* value	effect size
Age (years), median (IQR)	87 (81-90)	88 (85-92)	0.064	r = 0.15	87 (83-90)	86 (81-89)	0.341	r = 0.08
Sex, n (%)								
Male, n (%)	62 (52.5)	21 (50.0)	0.858	V = 0.02	41 (64.1)	41 (53.9)	0.234	V = 0.10
Female, n (%)	56 (47.5)	21 (50.0)			23 (35.9)	35 (46.1)		
Pre-admission residence, n (%)								
Home, n (%)	65 (55.1)	14 (33.3)	0.039	V = 0.19	43 (67.2)	28 (36.8)	0.001	V = 0.29
Nursing home, n (%)	51 (43.2)	27 (64.3)			20 (31.2)	44 (57.9)		
Hospital, n (%)	2 (1.7)	1 (2.4)			1 (1.6)	4 (5.3)		
Long-term care insurance system, n (%)								
Support need, n (%)	12 (10.2)	1 (2.4)	0.129	V = 0.17	10 (15.6)	7 (9.2)	0.04	V = 0.20
Long-term care, n (%)	96 (81.4)	40 (95.2)			45 (70.3)	66 (86.8)		
Aspiration risk conditions, n (%)	90 (76.3)	35 (83.3)	0.392	V = 0.08	48 (75.0)	52 (68.4)	0.455	V = 0.07
Stroke, n (%)	49 (41.5)	21 (50.0)	0.369	V = 0.08	24 (37.5)	28 (36.8)	1	V = 0.01
Neuromuscular disease, n (%)	17 (14.4)	5 (11.9)	0.799	V = 0.03	3 (4.7)	13 (17.1)	0.031	V = 0.18
Dementia, n (%)	56 (47.5)	18 (42.9)	0.719	V = 0.04	29 (45.3)	29 (38.2)	0.491	V = 0.07
History of AP, n (%)	9 (7.6)	5 (11.9)	0.524	V = 0.07	10 (15.6)	8 (10.5)	0.45	V = 0.07
A-DROP, median (IQR)	2 (2–3)	3 (2–4)	0.04	r = 0.16	2 (2–3)	2 (2–3)	0.308	r = 0.09
0 (Mild), n (%)	1 (0.8)	0 (0.0)			1 (1.6)	0 (0.0)		
1 (Moderate), n (%)	24 (20.3)	7 (16.7)			13 (20.3)	12 (15.8)		
2 (Moderate), n (%)	45 (38.1)	9 (21.4)			23 (35.9)	28 (36.8)		
3 (Severe), n (%)	31 (26.3)	15 (35.7)			20 (31.2)	24 (31.6)		
4 (Very severe), n (%)	14 (11.9)	11 (26.2)			7 (10.9)	10 (13.2)		
5 (Very severe), n (%)	3 (2.5)	0 (0.0)			0 (0.0)	2 (2.6)		
GNRI, mean ± SD	87.6 (10.9)	85.3 (11.9)	0.244	r = 0.09	87.0 (11.3)	85.0 (11.5)	0.316	r = 0.09
No risk, n (%)	23 (19.5)	7 (16.7)			14 (21.9)	10 (13.2)		
Low risk, n (%)	21 (17.8)	5 (11.9)			9 (14.1)	9 (11.8)		
Moderate risk, n (%)	38 (32.2)	13 (31.0)			18 (28.1)	25 (32.9)		
Major risk, n (%)	36 (30.5)	17 (40.5)			23 (35.9)	32 (42.1)		
Total FIM at admission, median (IQR)	30 (20-45)	23 (18–28)	0.001	r = 0.26	36 (24-53)	24 (20–32)	<0.001	r = 0.36
Motor FIM, median (IQR)	17 (13–29)	13 (13–15)	0.001	r = 0.27	17 (13-36)	13 (13–16)	<0.001	r = 0.35
Cognitive FIM, median (IQR)	12 (6–18)	9 (5–1)	0.013	r = 0.20	15 (10–19)	9 (7–14)	<0.001	r = 0.33
FOIS before onset, median (IQR)	7 (5–7)	6 (4–7)	0.123	r = 0.12	7 (6–7)	6 (4–7)	0.018	r = 0.20
Level 4, n (%)	23 (19.5)	13 (31.0)			8 (12.5)	21 (27.6)		
Level 5, n (%)	9 (7.6)	2 (4.8)			2 (3.1)	6 (7.9)		
Level 6, n (%)	26 (22.0)	11 (26.2)			18 (28.1)	18 (23.7)		
Level 7, n (%)	60 (50.8)	16 (38.1)			36 (56.2)	31 (40.8)		

Data are presented as mean (SD), median (IQR), frequency (%).

Abbreviations: ECPS, enhanced collaboration of physicians and speech-language therapists; AP, aspiration pneumonia; A-DROP, age, dehydration, respiratory failure, orientation disturbance, low blood pressure; GNRI, Geriatric Nutritional Risk Index; FIM, Functional Independence Measure; FOIS, Functional Oral Intake Scale

In the ECPS group, patients who achieved early oral intake were more likely to live at home prior to admission (55.1% vs. 33.3%; *p* = 0.039, V = 0.19), had lower A-DROP scores (2, IQR: 2–3 vs. 3, IQR: 2–4; *p* = 0.04, r = 0.16) and had higher total FIM scores (30, IQR: 20–45 vs. 23, IQR: 18–28; *p* = 0.001, r = 0.26).

In the control group, patients who achieved early oral intake were more likely to live at home prior to admission (67.2% vs. 36.8%; *p* = 0.001, V = 0.29), were less likely to be long-term care recipients (70.3% vs. 86.8%; *p* = 0.04, V = 0.20), and were less likely to have neuromuscular disease (4.7% vs. 17.1%; *p* = 0.031, V = 0.18). They also had higher total FIM scores (36, IQR: 24–53 vs. 24, IQR: 20–32; *p* < 0.001, r = 0.36), and had higher FOIS scores (7, IQR: 6–7 vs. 6, IQR: 4–7; *p* = 0.018, r = 0.20).

### Comparison of clinical outcomes between patients with and without early oral intake (within 2 days)

The results of comparison of clinical outcomes between patients with and without early oral intake are shown in Table 4. In the ECPS group, patients who achieved early oral intake had a shorter duration of antibiotic therapy (5, IQR: 4–6 vs. 6, IQR: 5–7; *p* = 0.036, r = 0.17), shorter length of hospital stay (12, IQR: 8–18 vs. 18, IQR: 13–30; *p* < 0.001, r = 0.31), and lower rates of change of residence (28.0% vs. 47.6%; *p* = 0.023, V = 0.18). There were no significant differences in the resumption of antibiotic therapy, decline in FOIS, or the use of artificial nutrition at discharge.

**Table 4:** Comparison of clinical outcomes between patients with and without early oral intake (within 2 days).

	ECPS group				Control group			
	early oral intake n = 118	non-early oral intake n = 42	*p* value	effect size	early oral intake n = 64	non-early oral intake n = 76	*p* value	effect size
**Post-admission process variables**								
Time to SLT assessment, median (IQR)	1 (1–1)	2 (1–3)	<0.001	r = 0.36	1 (1–2)	2 (1–3)	0.072	r = 0.15
Time to oral intake initiation, median (IQR)	1 (1–2)	4 (3–4)	<0.001	r = 0.79	1 (1–2)	4 (3–5)	<0.001	r = 0.88
**Clinical outcomes**								
Duration of antibiotic therapy, median (IQR)	5 (4–6)	6 (5–7)	0.036	r = 0.17	7 (6–11)	8 (6–10)	0.602	r = 0.04
Resumption of antibiotic therapy, n (%)	6 (5.1)	3 (7.1)	0.699	V = 0.04	7 (10.9)	16 (21.1)	0.117	V = 0.13
Length of hospital stay, median (IQR)	12 (8–18)	18 (13-30)	<0.001	r = 0.31	15 (10-28)	21 (14-33)	0.008	r = 0.23
Total FIM at discharge, median (IQR)	36 (21-58)	25 (18-37)	0.014	r = 0.19	47 (28-67)	28 (20-44)	<0.001	r = 0.36
Motor FIM, median (IQR)	20 (13-42)	14 (13–20)	0.013	r = 0.20	30 (17-49)	15 (13–29)	<0.001	r = 0.36
Cognitive FIM, median (IQR)	13 (7–19)	10 (5–15)	0.051	r = 0.15	17 (10–21)	11 (7–16)	0.001	r = 0.28
Motor FIM gain, median (IQR)	0 (0-8)	0 (0-4)	0.445	r = 0.06	5 (0-16)	0 (0-6)	0.023	r = 0.19
Cognitive FIM gain, median (IQR)	0 (0−0)	0 (0−0)	0.858	r = 0.01	0 (0−0)	0 (0−0)	0.89	r = 0.01
Post-discharge residence, n (%)								
Home, n (%)	46 (39.0)	7 (16.7)	0.017	V = 0.22	30 (46.9)	16 (21.1)	0.004	V = 0.26
Nursing home, n (%)	45 (38.1)	19 (45.2)			22 (34.4)	34 (44.7)		
Hospital, n (%)	27 (22.9)	16 (38.1)			12 (18.8)	26 (34.2)		
Change of residence, n (%)	33 (28.0)	20 (47.6)	0.023	V = 0.18	17 (26.6)	27 (35.5)	0.278	V = 0.09
FOIS at discharge, median (IQR)	5 (4–6)	4 (4–5)	0.005	r = 0.22	5 (4–6)	4 (2–5)	0.002	r = 0.27
Level 1, n (%)	5 (4.2)	3 (7.1)			4 (6.2)	12 (15.8)		
Level 2, n (%)	5 (4.2)	5 (11.9)			2 (3.1)	8 (10.5)		
Level 3, n (%)	1 (0.8)	1 (2.4)			0 (0.0)	0 (0.0)		
Level 4, n (%)	32 (27.1)	15 (35.7)			13 (20.3)	20 (26.3)		
Level 5, n (%)	36 (30.5)	12 (28.6)			24 (37.5)	22 (28.9)		
Level 6, n (%)	19 (16.1)	3 (7.1)			12 (18.8)	12 (15.8)		
Level 7, n (%)	20 (16.9)	3 (7.1)			9 (14.1)	2 (2.6)		
Decline in FOIS, n (%)	70 (59.3)	30 (71.4)	0.196	V = 0.11	46 (71.9)	59 (77.6)	0.442	V = 0.06
Artificial nutrition at discharge, n (%)	11 (9.3)	9 (21.4)	0.056	V = 0.16	6 (9.4)	20 (26.3)	0.015	V = 0.20

Data are presented as median (IQR) or frequency (%). Variables in this table were recorded after initiation of management and represent the clinical course and outcomes during hospitalization and at discharge.

Abbreviations: ECPS, enhanced collaboration of physicians and speech-language therapists; SLT, Speech-Language Therapist; FIM, Functional Independence Measure; FOIS, Functional Oral Intake Scale

In the control group, patients who achieved early oral intake had a shorter length of hospital stay (15, IQR: 10–28 vs. 21, IQR: 14–33; *p* = 0.008, r = 0.23), and a lower rate of artificial nutrition (9.4% vs. 26.3%; *p* = 0.015, V = 0.20). There were no significant differences in the duration of antibiotic therapy, the resumption of antibiotic therapy, change of residence, or decline in FOIS.

### Multivariate logistic regression analysis for early oral intake (within 2 days)

Among the 300 participants included in the analysis, 182 achieved oral intake within two days and 118 did not. The final multivariable model included 11 predictor variables, yielding an EPV of 10.7 (118/11), thus exceeding the commonly recommended minimum of 10. Several factors were associated with early oral intake (Table 5). Pre-admission residence (OR = 0.54, 95% CI: 0.31–0.92, *p* = 0.023) was a factor, indicating that patients residing outside the home had about 46% lower odds of achieving early oral intake. Aspiration risk conditions were a factor, (OR = 2.33, 95% CI: 1.15–4.70, *p* = 0.018), suggesting more than double the odds of early oral intake. Total FIM at admission (OR = 1.04, 95% CI: 1.01–1.06, *p* < 0.005) was a factor, which indicates a 4% increase in odds per unit increase in FIM. Time to SLT assessment was a factor, (OR = 0.69, 95% CI: 0.53–0.90, *p* < 0.01) reflecting a 31% decrease in odds with delayed assessment. Finally, ECPS was a factor (OR = 3.85, 95% CI: 2.22–6.70, *p* < 0.001), indicating nearly fourfold increased odds of early oral intake.

**Table 5:** Multivariate logistic regression analysis for early oral intake (within 2 days).

	Odds ratio	95% CI	*p* value
Age	0.99	0.96-1.03	0.770
Sex	1.21	0.68-2.17	0.510
Pre-admission residence	0.54	0.31-0.92	0.023
Long-term care insurance system	0.65	0.36-1.19	0.160
Aspiration risk conditions	2.33	1.15-4.70	0.018
A-DROP	0.90	0.68-1.20	0.480
GNRI	1.00	0.97-1.03	0.940
Total FIM at admission	1.04	1.01-1.06	< 0.005
FOIS before onset	1.07	0.83-1.38	0.600
Time to SLT assessment	0.69	0.53-0.90	< 0.01
ECPS	3.85	2.22-6.70	< 0.001

Explanatory variables: Age, Sex (reference: male), Pre-admission residence (reference: home), Long-term care insurance system (reference: non-use), Aspiration risk conditions (reference: absent), A-DROP, GNRI, Total FIM at admission, FOIS before onset, Time to SLT assessment, ECPS (reference: absent).

Abbreviations: A-DROP, age, dehydration, respiratory failure, orientation disturbance, low blood pressure; GNRI, Geriatric Nutritional Risk Index; FIM, Functional Independence Measure; FOIS, Functional Oral Intake Scale; SLT, Speech-Language Therapist; ECPS, Enhanced collaboration of physicians and speech-language therapists

## Discussion

We examined the impact of structured collaboration between physicians and SLTs on acute care for older adults with AP, focusing on early oral intake (within two days). Implementation of ECPS was strongly associated with early oral intake. Additionally, delayed swallowing assessment by SLTs was negatively associated with early oral intake, which underscores the importance of timely swallowing assessment. Implementation of this structured inter-professional clinical pathway is suggested by these results to influence the timing of oral intake in this population. Importantly, both ECPS and the timing of swallowing assessment are modifiable clinical factors, representing actionable targets for facilitating early oral intake in clinical practice.

An exploratory analysis of safety endpoints suggested that early oral intake was not associated with any clear increase in disadvantages, including duration of antibiotic therapy, resumption of antibiotics due to recurrence of AP, change in residence, decline in swallowing function, need for artificial nutrition at discharge, or length of hospital stay. Although these findings are based on an exploratory analysis, they may provide preliminary insights for considering early oral intake in older adults with AP. Taken together, these findings highlight the potential value of structured inter-professional collaboration in promoting early oral intake in older adults with AP.

Although ECPS implementation and shorter time to SLT assessment were associated with early oral intake initiation, not all institutions may be able to implement such structured systems because of manpower or facility constraints. Our findings suggest that several patient-related factors may serve as practical reference points even outside an ECPS framework. Higher FIM at admission was independently associated with early oral intake initiation, suggesting that preserved functional independence may facilitate earlier resumption of oral intake. In addition, patients admitted from home were more likely to initiate oral intake early, possibly reflecting better premorbid functional reserve. Interestingly, the presence of aspiration risk conditions was also associated with early initiation, which may reflect increased clinical vigilance and prioritization of swallowing assessment in patients perceived to be at higher risk. Although decisions regarding oral intake should be based on careful swallowing assessment, these factors may provide useful clinical information when considering early oral intake, particularly in settings where an ECPS system is not available.

In the ECPS group and in patients who achieved early oral intake, reductions were observed in resumption of antibiotic therapy, length of hospital stay, and duration of antimicrobial therapy. SLTs do not directly determine antimicrobial management, so it is unlikely that the intervention directly influenced antibiotic duration. However, international clinical nutrition guidelines recommend initiation of enteral nutrition within 24–48 hours in acutely ill patients with a functional gastrointestinal tract. Early nutritional support has been associated with reduced infectious complications and improved clinical outcomes [33,34]. Although direct evidence regarding early oral feeding in aspiration pneumonia is limited, the physiological rationale underlying early nutritional support may help to explain our results.

Another notable observation was that in the control group, neuromuscular disease, higher long-term care needs, and lower FOIS scores showed significant differences in relation to the timing of oral intake, but this was not the case in the ECPS group. Neuromuscular disease is a well-established risk factor for dysphagia, and higher care needs as well as lower FOIS levels reflect greater frailty and impaired swallowing function. In the control group, clinicians may have been more cautious in initiating oral intake in patients with these characteristics due to the perceived risk of aspiration. In contrast, in the ECPS group, decisions were guided by standardized swallowing assessments and predefined criteria. This finding may suggest that the ECPS approach helped to facilitate earlier oral intake decisions based on objective swallowing assessments rather than baseline clinical characteristics alone.

From a swallowing rehabilitation perspective, early oral intake offers multiple benefits. A tentative nil per os regimen reportedly impairs swallowing abilities in patients with AP [30]. Conversely, early oral intake increases the likelihood of oral intake at discharge [15,16] and it decreases reliance on artificial nutrition [17]. In our cohort, patients were generally of advanced age, had low ADL levels and poor nutritional status, and many presented with moderate-to-severe pneumonia. There was usually minimal change in their ADLs. In such circumstances, opportunities for active functional recovery are limited, and maintaining swallowing function becomes particularly important. Without food intake, SLTs typically employ indirect swallowing exercises, which are often impractical for patients to perform intensively. Oral intake, however, provides direct swallowing exercise. We think early swallowing assessment and timely introduction of oral feeding, accompanied by appropriate risk management, allow for increased opportunities for functional swallowing practice. This approach aligns with the core principles of the McNeil Dysphagia Therapy Program [35], which emphasizes the use of real-food swallowing tasks to facilitate recovery. Additionally, stimulating salivary flow is said to be important for maintaining oral health [36], and food intake can influence circadian rhythm [37]. Stabilizing circadian rhythm also improves activity levels, reducing the risk of missed rehabilitation opportunities. Immobility and being bedridden are well-recognized risk factors for AP [2–4]. Early oral intake therefore likely helps to preserve swallowing function in older adult patients with AP by considering the interconnection of various factors. Moreover, the nutritional status of patients with AP is a predictor of their prognosis [38], and insufficient energy intake in the first week following admission is a risk factor for adverse outcomes [39]. Nutritional management in patients with pneumonia is associated with a more favorable prognosis [40,41]. To optimize nutritional management in older adults with AP, we suggest the importance of early consideration of swallowing assessment and oral intake. Decisions regarding artificial nutrition for older adults with AP should be made through a careful and thorough process that provides comprehensive information to patients, their families, and healthcare professionals about the potential benefits and risks [42,43]. Decision-making often requires time, so prompt and user-friendly information on swallowing function is particularly important. Early assessment of swallowing and consideration of oral feeding are key elements that facilitate this process.

Objective and generalizable criteria for resuming oral intake in patients with AP remain undefined [20]. Importantly, patients vary in terms of age, living environment, comorbidities, and the specific preferences and requests from both the patient and their family regarding care. Additionally, there is considerable variability in patients’ current physical and swallowing function, which may contribute to the difficulty in identifying objective criteria for resuming oral intake. Considering the multiple factors involved in swallowing function, nutritional management, and decision-making support, early consideration of oral intake may serve as a pivotal starting point in the management of older adults with AP. However, we do not advocate indiscriminate implementation of early oral intake; careful risk management and interdisciplinary collaboration are still essential. Enhanced collaboration and mutual understanding between physicians and swallowing specialists regarding early oral intake could represent one factor worth considering in achieving effective management in older adults with AP.

This study has several limitations that warrant mention. Firstly, due to its retrospective cohort design, causal relationships cannot be definitively established. A randomized controlled trial would be preferable for assessing the efficacy of ECPS and early oral intake.

Also, it was conducted at a single facility, the data were relatively old, and some selection bias is inevitable, which limits generalizability. Additionally, the exclusion of deceased patients and those with severe dysphagia means the findings may not be applicable to all patients with AP. The effectiveness of ECPS and early oral intake should therefore be assessed in multi-center studies.

Furthermore, because this study used a before–after design comparing patients admitted before and after April 2017, we cannot exclude the possibility of time-related confounding. Changes in institutional policies, staffing, clinical awareness, or broader trends in the management of pneumonia over time may have influenced the observed differences between the groups.

Moreover, secondary outcomes were derived from exploratory analyses with limited adjustment for potential confounders. The results should therefore be interpreted as hypothesis-generating, and further prospective studies are warranted to confirm the safety and clinical benefits of early oral intake in elderly patients with aspiration pneumonia.

Another consideration is that the timing of SLT initiation was recorded based on hospital calendar days rather than the exact elapsed time from admission. The day of admission was defined as day 0 regardless of the actual admission time. Patients classified as receiving SLT initiation on the same hospital day may therefore have differed by several hours in actual elapsed time. The observed statistical differences between groups may not fully reflect differences at the hour level, and the clinical significance of small differences within a single calendar day should be interpreted with caution. Future studies incorporating more precise time-to-intervention data would help clarify the clinical impact of early SLT initiation.

Finally, the appropriateness of the subjects’ pre-illness diets in relation to their swallowing function remains unclear, as many patients were on diets that were not dysphagia-friendly. Pre-illness assessments are necessary to better understand swallowing function change. Additionally, reliable data sharing across medical institutions would improve the accuracy of variable adjustments.

## Conclusion

The implementation of a structured inter-professional clinical pathway facilitating ECPS was associated with earlier initiation of oral intake in our cohort. Early oral intake showed no indication of an increase in any of the disadvantages. Such collaboration could represent an important factor in achieving effective management in older adults with AP.

## Supporting information

S1 Data Minimal dataset underlying the findings of this study. (XLSX)

## Abbreviations

ADL: activities of daily living
A-DROP: age, dehydration, respiratory failure, orientation disturbance, low blood pressure
AP: aspiration pneumonia
CURB-65: confusion, urea nitrogen, respiratory rate, blood pressure, age ≥65 years
ECPS: enhanced collaboration of physicians and speech-language therapists
FIM: functional independence measure
FOIS: functional oral intake scale
GNRI: geriatric nutritional risk index
SLT: speech-language therapist
