# Supplementary material for: Enhanced collaboration of physicians and speech-language therapists promotes early oral intake in older adults with aspiration pneumonia: A retrospective cohort study
Source: PLoS One. 2026 Jun 10;21(6):e0350495. doi: 10.1371/journal.pone.0350495 (PMC13252771; doi:10.1371/journal.pone.0350495)
Supplement: Attachment — Submitted filename: renamed_f9540.docx [file pone.0350495.s002.docx]

This study presents the interesting finding that active intervention by speech-language therapists (SLTs) in patients with aspiration pneumonia may facilitate early oral intake and be associated with subsequent patient outcomes. Against the backdrop of increasing aspiration pneumonia patients in Japan, this study addresses an important theme. However, the writing is generally redundant and requires appropriate revisions, including improvements to its structure.

**Major comments**

1. The fundamental differences between the ECPS group and the control group are unclear. The decision-making process within the ECPS group should be described more specifically. Is it correct to understand that the difference between the two groups lies solely in who decides the timing of initiating oral intake? Were diet orders for both groups placed by physicians? The meaning of “a diet order was placed simultaneously with the referral to the SLT (Page 8, line 184-185)” is unclear. Additionally, clarification is needed on whether there were substantive differences between the groups in subsequent rehabilitation content or interventions.

However, the paragraph on “Dysphagia rehabilitation system at our hospital” is somewhat redundant; it would be preferable to reorganize and summarize it for clarity.

2. SLT initiation generally occurred within 1 (1-2) days in both the ECPS and control groups. A statistically significant difference existed between the groups, and the authors demonstrated, based on multivariate analysis, that delayed SLT initiation delayed oral intake initiation by 31%. However, questions remain regarding the clinical significance of this “difference within one day (or a matter of hours).” For example, in sepsis, the importance of initiating antimicrobial therapy within one hour of diagnosis is debated. Similarly, in this study, it requires consideration of whether the intervention at the time unit level demonstrates clinical utility.
3. In the ECPS group and the early oral intake group, reductions were observed in “resumption of antibiotic therapy,” “length of hospital stay,” and “duration of antimicrobial therapy.” While it is unlikely that SLT directly influenced the duration of antimicrobial administration, consideration is warranted regarding the potential impact of early initiation of oral intake on these outcomes.
4. The entire section in Discussion is redundant, with the second ”Multivariate analysis…” and third “Our exploratory analysis…” paragraphs largely consisting of restating the results. It would be preferable to concisely summarize these points and then logically transition to the discussion in the fourth paragraph “From a swallowing…”.
5. While the importance of collaboration with SLTs and physicians is understood, it is anticipated that some medical institutions may be unable to establish similar systems due to manpower or facility constraints. Based on the results of this study, please briefly discuss the patient backgrounds and clinical factors that could serve as reference points when considering initiating oral intake outside of ECPS.

**Minor comments**

1. The “Study design and setting” section lists the study period and data access period, while the ‘Exposure’ section indicates the patient enrollment period. However, these descriptions are scattered and difficult to understand. It is desirable to clarify why the study period and data access period differ, and to consolidate the description including the patient enrollment period into the “Study design and setting” section.
2. Page 6, line 120

“…and upper respiratory tract symptoms such as sputum production”

Sputum production may correspond to “lower” respiratory tract symptoms.

3. Page 9, line 189-191

“In contrast, patients in the control group… and the timing of swallowing assessment and oral intake was determined at the discretion of the attending physician”

The statement in the introduction that “Even before the implementation of this protocol, SLTs at our hospital routinely conducted swallowing assessment at an early stage” appears contradictory. The wording needs to be revised.

4. Page 9, line 200

“…defined as before as the initiation of oral intake within two days after admission”

“as before” seems grammatically inappropriate.

5. While episodes of aspiration are useful evidence for suspecting aspiration pneumonia, please clarify the reason for excluding “patients with AP due to vomiting” in the exclusion criteria.
6. Additional explanation should be provided regarding the rationale for selecting the factors used as explanatory variables in the multivariate logistic regression analysis within the statistical analysis.
7. In the Results section, the independent presentation of “Exclusion criteria” may be confusing to readers. Presenting the number and reasons for excluded cases as part of the “Baseline patient characteristics” could help improve the overall clarity and readability of the Results.
8. Page 29, line 421-423

The statement “Our patients with AP, who … in their ADLs” within the Discussion section feels abrupt. Please clarify the intent of this sentence.

9. It is interesting that “neuromuscular disease” acts as an inhibitor of early oral intake in the control group. Regarding the absence of a similar effect in the ECPS group, please add a brief discussion on whether this can be interpreted as an effect of the ECPS intervention.
