# Supplementary material for: Enhanced collaboration of physicians and speech-language therapists promotes early oral intake in older adults with aspiration pneumonia: A retrospective cohort study
Source: PLoS One. 2026 Jun 10;21(6):e0350495. doi: 10.1371/journal.pone.0350495 (PMC13252771; doi:10.1371/journal.pone.0350495)
Supplement: Attachment — Submitted filename: Review tazawa.docx [file pone.0350495.s003.docx]

**Overall assessment**

This study evaluates the association between the implementation of enhanced collaboration between physicians and speech-language therapists (ECPS) and early initiation of oral intake in older adults with aspiration pneumonia. The dataset is substantial, and the statistical analyses are generally appropriate. The findings are clinically interesting. With clarification and refinement in several key areas—particularly regarding the definition and positioning of ECPS—the manuscript has the potential to become a valuable contribution.

**Major Comments**

**1. Clarification of ECPS Intervention (Primary Concern)**

The most important issue concerns the description of ECPS.

Although the study demonstrates a statistically significant association between ECPS implementation and early oral intake without an apparent increase in adverse outcomes, the specific procedural and structural elements of ECPS remain insufficiently described.

Terms such as “shared understanding” and “organized workflow” are abstract and do not allow readers to understand what concrete changes were implemented in daily clinical practice.

From the Introduction, it appears that SLTs were already performing early swallowing assessments prior to ECPS implementation, and that delays primarily occurred at the stage of physician decision-making. If so, ECPS may represent a standardization and streamlining of physician decision-making processes rather than an enhancement of interdisciplinary rehabilitation itself.

To improve transparency and reproducibility, please clarify:

- What specific procedural changes were introduced in physician ordering processes
- Whether predefined criteria for oral intake initiation were established
- How decision-making authority differed before and after ECPS
- What concrete elements distinguish ECPS from prior usual care

A clearer description would allow readers to understand whether ECPS represents enhanced collaboration, care pathway redesign, or decision-making standardization.

**2. Conceptual Framing of the Research Question**

Because ECPS was explicitly designed to facilitate earlier initiation of oral intake, and early oral intake was selected as the primary outcome, the research question appears somewhat self-contained.

In other words, the intervention was structured to accelerate oral intake, and the outcome measures acceleration of oral intake. While this does not invalidate the study, clarifying that the manuscript evaluates the effectiveness of a standardized care pathway intended to accelerate decision-making—rather than demonstrating rehabilitative efficacy per se—would strengthen the conceptual framing.

Repositioning the study as an evaluation of a structured clinical pathway may enhance interpretability.

**3. Inconsistencies in Study Period Description**

There are inconsistencies in the description of the study period across sections of the manuscript.

- The Study Design section describes the study as conducted between October 2019 and March 2022.
- The Exposure section refers to a period from February 2015 to February 2019.
- Data access dates are also described separately.

These discrepancies create confusion regarding the actual patient inclusion period and the timeframe used for group allocation. Because the exposure definition is directly linked to calendar time (before vs. after April 2017), precise specification of the study period is critical.

Please clarify:

- The exact patient inclusion period
- The timeframe used for exposure classification
- The relationship between data access dates and study period

Consistency across all sections is essential.

**4. Time-Related Confounding (Before–After Design)**

The study compares patients admitted before and after April 2017. Although understandable, this design inherently introduces potential time-related confounding.

Changes in institutional policy, staffing, clinical awareness, or broader trends in pneumonia management over time may have contributed to the observed differences. This limitation should be discussed more explicitly.

Additionally, the rationale for the chosen cutoff date requires clearer justification.

**5. Definition of Early Oral Intake**

The phrase “defined as before as the initiation of oral intake within two days after admission” should be revised for clarity.

Furthermore, defining early oral intake as “within two days after admission” may lead to substantial time differences depending on admission timing (e.g., daytime vs. nighttime admission). Please clarify whether this was defined by calendar days or a fixed 48-hour window, and whether this issue was considered in the analysis.

**6. Operational Definitions of Secondary Outcomes**

Some secondary outcomes (e.g., prolonged antibiotic therapy, extended hospitalization) are not operationally defined. Please clarify how these outcomes were defined or acknowledge this as a limitation.

**7. Pre-Onset FOIS Determination**

The method used to determine the pre-onset FOIS score requires further clarification. Please specify:

- The information sources used
- Who performed the assessment
- Whether a standardized mapping rule was used

This is important for reproducibility.

**8. Organization of Methods**

There is some overlap between the Outcome section and the “Measurements of covariates” section, and variables with different analytical roles are intermingled.

Reorganizing the Methods to clearly distinguish:

- Baseline covariates
- Primary outcome
- Secondary outcomes
- Post-admission process variables

would improve clarity and reproducibility.

**9. Sample Size and EPV**

The sample size calculation assumes 10 predictor variables; however, the final multivariable model appears to include more variables.

Please clarify:

- The final number of predictors included
- The total number of outcome events
- Whether the events-per-variable criterion was satisfied based on the actual model

**Minor Comments**

- Improve the clarity and resolution of Figures so that the workflow differences between groups are more clearly illustrated.
- Revise minor grammatical issues for clarity.

**Final Recommendation**

This is a well-conducted retrospective analysis with clinically relevant findings. The primary area requiring improvement is the clearer specification and positioning of the ECPS intervention, along with clarification of study period inconsistencies. Addressing these issues would substantially strengthen the manuscript.
