# Supplementary material for: Enhanced collaboration of physicians and speech-language therapists promotes early oral intake in older adults with aspiration pneumonia: A retrospective cohort study
Source: PLoS One. 2026 Jun 10;21(6):e0350495. doi: 10.1371/journal.pone.0350495 (PMC13252771; doi:10.1371/journal.pone.0350495)
Supplement: Attachment — Submitted filename: Response to Reviewers.docx [file pone.0350495.s004.docx]

Dear Editor,

We sincerely thank you and the reviewers for your valuable comments and suggestions, which have helped us improve the quality of our manuscript.

We have carefully revised the manuscript accordingly. Our detailed responses are provided below.

--------------------------------

Response to the Editor

--------------------------------

**Comment 1:**

Please ensure that your manuscript meets PLOS ONE's style requirements, including those for file naming. The PLOS ONE style templates can be found at

https://journals.plos.org/plosone/s/file?id=wjVg/PLOSOne_formatting_sample_main_body.pdf and

https://journals.plos.org/plosone/s/file?id=ba62/PLOSOne_formatting_sample_title_authors_affiliations.pdf

**Response:**

Thank you for your comment. We have checked the manuscript to ensure that it complies with PLOS ONE's style requirements, including file naming, according to the provided templates.

**Comment 2:**

We note that your Data Availability Statement is currently as follows: All relevant data are within the manuscript and in Supporting Information files.

Please confirm at this time whether or not your submission contains all raw data required to replicate the results of your study. Authors must share the “minimal data set” for their submission. PLOS defines the minimal data set to consist of the data required to replicate all study findings reported in the article, as well as related metadata and methods (https://journals.plos.org/plosone/s/data-availability#loc-minimal-data-set-definition).

For example, authors should submit the following data:

- The values behind the means, standard deviations and other measures reported;

- The values used to build graphs;

- The points extracted from images for analysis.

Authors do not need to submit their entire data set if only a portion of the data was used in the reported study.

If your submission does not contain these data, please either upload them as Supporting Information files or deposit them to a stable, public repository and provide us with the relevant URLs, DOIs, or accession numbers. For a list of recommended repositories, please see https://journals.plos.org/plosone/s/recommended-repositories.

If there are ethical or legal restrictions on sharing a de-identified data set, please explain them in detail (e.g., data contain potentially sensitive information, data are owned by a third-party organization, etc.) and who has imposed them (e.g., an ethics committee). Please also provide contact information for a data access committee, ethics committee, or other institutional body to which data requests may be sent. If data are owned by a third party, please indicate how others may request data access.

**Response:**

Thank you for your comment. We confirm that all raw data required to replicate the results of this study are provided in the Supporting Information files. The minimal data set underlying the findings has been prepared and uploaded as S1 Data. We have also revised the Data Availability Statement in the manuscript accordingly (Page 35, Lines 558–560).

**Comment 3:**

When completing the data availability statement of the submission form, you indicated that you will make your data available on acceptance. We strongly recommend all authors decide on a data sharing plan before acceptance, as the process can be lengthy and hold up publication timelines. Please note that, though access restrictions are acceptable now, your entire data will need to be made freely accessible if your manuscript is accepted for publication. This policy applies to all data except where public deposition would breach compliance with the protocol approved by your research ethics board. If you are unable to adhere to our open data policy, please kindly revise your statement to explain your reasoning and we will seek the editor's input on an exemption. Please be assured that, once you have provided your new statement, the assessment of your exemption will not hold up the peer review process.

**Response:**

Thank you for your comment. We have revised the Data Availability Statement to clarify that all relevant data are available within the manuscript and its Supporting Information files (S1 Data). Therefore, the data are fully accessible without restriction.

**Comment 4:**

Please amend the manuscript submission data (via Edit Submission) to include author Atsushi Keyaki.

**Response:**

Thank you for your comment. We have amended the manuscript submission data via Edit Submission to include the author Atsushi Keyaki.

**Comment 5:**

If the reviewer comments include a recommendation to cite specific previously published works, please review and evaluate these publications to determine whether they are relevant and should be cited. There is no requirement to cite these works unless the editor has indicated otherwise.

**Response:**

Thank you for your comment. We confirm that no specific recommendations to cite additional publications were provided by the reviewers, and therefore no changes were made.

--------------------------------

Response to Reviewer #1

--------------------------------

**Major comments**

**Comment 1:**

The fundamental differences between the ECPS group and the control group are unclear. The decision-making process within the ECPS group should be described more specifically. Is it correct to understand that the difference between the two groups lies solely in who decides the timing of initiating oral intake? Were diet orders for both groups placed by physicians? The meaning of “a diet order was placed simultaneously with the referral to the SLT (Page 8, lines 184-185)” is unclear. Additionally, clarification is needed on whether there were substantive differences between the groups in subsequent rehabilitation content or interventions.

However, the paragraph on “Dysphagia rehabilitation system at our hospital” is somewhat redundant; it would be preferable to reorganize and summarize it for clarity.

**Response:**

Thank you for this important and insightful comment. We agree that the description of the intervention was not sufficiently clear. We have therefore clarified the differences between the ECPS and control groups in the Exposure subsection of the Methods of the revised manuscript (Pages 7-8, lines 161-183).

In the ECPS group, the intervention consisted of three components:

(1) an inter-professional agreement supporting early swallowing assessment and oral intake,

(2) a standardized management protocol integrated into the aspiration pneumonia treatment protocol, and

(3) a coordinated clinical workflow between physicians and SLTs.

In contrast, in the control group, decisions regarding swallowing assessment and initiation of oral intake were made at the discretion of individual attending physicians, without a predefined inter-professional agreement, standardized protocol, or coordinated workflow.

We have also clarified that diet orders in both groups were placed by physicians (Page 3, lines 71-73). Under Japanese law, SLTs provide dysphagia-related care under physician supervision, and both swallowing assessment and initiation of oral intake require a physician’s authorization.

In addition, we have revised the description regarding the simultaneous diet order and SLT referral for clarity (Page 8, lines 173-175). The revised text reads as follows:

“Physicians ordered a swallowing assessment from a SLT upon admission and entered diet orders in advance if deemed clinically appropriate.”

Furthermore, there were no substantive differences in dysphagia rehabilitation between the two groups during the inclusion period. ECPS reorganized and standardized the clinical workflow and timing of decisions, while there were no changes to the decision-making authority, the clinical criteria used by SLTs to assess eligibility for oral intake, and the content of dysphagia rehabilitation. We have clarified this point in the revised manuscript (Page 8, lines 184-187).

Regarding the reviewer’s comment on the “Dysphagia rehabilitation system” section, we agree that the original description was somewhat redundant. We have reframed and carefully revised the section with the revised title of ‘Our dysphagia rehabilitation system” to improve clarity (Pages 6-7, lines 129-150). We think there is genuine benefit of describing our system so that our findings can be understood in context.

**Comment 2:**

SLT initiation generally occurred within 1 (1-2) days in both the ECPS and control groups. A statistically significant difference existed between the groups, and the authors demonstrated, based on multivariate analysis, that delayed SLT initiation delayed oral intake initiation by 31%. However, questions remain regarding the clinical significance of this “difference within one day (or a matter of hours).” For example, in sepsis, the importance of initiating antimicrobial therapy within one hour of diagnosis is debated. Similarly, in this study, it requires consideration of whether the intervention at the time unit level demonstrates clinical utility.

**Response:**

We thank the reviewer for raising this important question regarding the clinical significance of the observed difference in SLT initiation timing.

In our dataset, hospital day was categorized based on calendar days, with the day of admission defined as day 0 regardless of the exact admission time. Patients classified as having SLT initiation on the same hospital day may therefore have differed by several hours in actual elapsed time from admission. As a result, the statistically significant difference observed between groups at the calendar-day level may not fully reflect differences at the hour level.

We agree that the clinical significance of a difference within one calendar day may appear modest when considered in isolation. However, in the clinical workflow of dysphagia management for aspiration pneumonia, we suggest that swallowing assessment by an SLT is a key step that precedes physician decision-making regarding the initiation of oral intake. Even a delay of several hours or within a single calendar day may influence the timing of oral intake initiation in practice.

Additionally, the upper range of the interquartile distribution was truncated in the categorical presentation, and this may have contributed to the statistical significance.

Unfortunately, in the present study we did not extract admission time data, so a more granular time-based analysis was not feasible. We have added this point to the Limitations section (Pages 32–33, Lines 510–518), acknowledging that future studies incorporating precise time-to-intervention data are needed to better clarify the clinical utility of early SLT initiation.

**Comment 3:**

In the ECPS group and the early oral intake group, reductions were observed in “resumption of antibiotic therapy,” “length of hospital stay,” and “duration of antimicrobial therapy.” While it is unlikely that SLT directly influenced the duration of antimicrobial administration, consideration is warranted regarding the potential impact of early initiation of oral intake on these outcomes.

**Response:**

Thank you for this insightful comment. We agree that SLTs do not directly determine antimicrobial management. In response, we have revised the Discussion section to clarify this point and added a brief explanation regarding the potential role of early nutritional support, referring to international clinical nutrition guidelines. The relevant text has been added to the Discussion section (Page 29, Lines 425–434).

**Comment 4:**

The entire section in Discussion is redundant, with the second ”Multivariate analysis…” and third “Our exploratory analysis…” paragraphs largely consisting of restating the results. It would be preferable to concisely summarize these points and then logically transition to the discussion in the fourth paragraph “From a swallowing…”.

**Response:**

Thank you for this helpful suggestion. We agree that parts of the Discussion section were overly descriptive of the results. In response, we have condensed the second and third paragraphs to reduce redundancy and to provide a more concise summary of the main findings (Page 28, Lines 394–408). The revised text now transitions more directly to the subsequent discussion on clinical interpretation.

**Comment 5:**

While the importance of collaboration with SLTs and physicians is understood, it is anticipated that some medical institutions may be unable to establish similar systems due to manpower or facility constraints. Based on the results of this study, please briefly discuss the patient backgrounds and clinical factors that could serve as reference points when considering initiating oral intake outside of ECPS.

**Response:**

Thank you for this valuable suggestion. We have added a brief discussion on patient-related factors that may serve as practical reference points when considering early oral intake outside an ECPS framework. Specifically, we describe the potential relevance of admission FIM score, pre-admission living situation, and aspiration risk conditions, which were identified as independent factors associated with early oral intake in the multivariate analysis. The revised text has been added to the Discussion section (Pages 28-29, Lines 411–424).

**Minor comments**

**Comment 1:**

The “Study design and setting” section lists the study period and data access period, while the ‘Exposure’ section indicates the patient enrollment period. However, these descriptions are scattered and difficult to understand. It is desirable to clarify why the study period and data access period differ, and to consolidate the description including the patient enrollment period into the “Study design and setting” section.

**Response:**

Thank you for this helpful suggestion. We agree that it was confusing, so we have decided to remove the data access dates from the manuscript. We think this helps to clarify our intended meaning. We have reorganized the description of the study timeline by consolidating the patient enrollment period, study period into the “Study design and setting” section (Page 5, Lines 95–104).

**Comment 2:**

Page 6, line 120

“…and upper respiratory tract symptoms such as sputum production”

Sputum production may correspond to “lower” respiratory tract symptoms.

**Response:**

Thank you for pointing this out. We agree that sputum production is not specific to upper respiratory tract symptoms. We have revised the sentence accordingly to refer to “respiratory symptoms” instead (Page 5, Line 116).

**Comment 3:**

Page 9, line 189-191

“In contrast, patients in the control group… and the timing of swallowing assessment and oral intake was determined at the discretion of the attending physician”

The statement in the introduction that “Even before the implementation of this protocol, SLTs at our hospital routinely conducted swallowing assessment at an early stage” appears contradictory. The wording needs to be revised.

**Response:**

Thank you for this comment. We agree that the previous wording could appear inconsistent. To avoid confusion, we have removed the sentence that described routine early swallowing assessment by SLTs, and we have reorganized the section to focus on the structural components of ECPS and the differences between the ECPS and control groups.

**Comment 4:**

Page 9, line 200

“…defined as before as the initiation of oral intake within two days after admission”

“as before” seems grammatically inappropriate.

**Response:**

Thank you for your comment. The phrase “as before” has been removed, and the definition has been clarified (Page 10, Lines 219–223). Early oral intake is now operationally defined as the initiation of oral intake within two calendar days after admission, counting the day of admission as day 0 (days 0–2).

**Comment 5:**

While episodes of aspiration are useful evidence for suspecting aspiration pneumonia, please clarify the reason for excluding “patients with AP due to vomiting” in the exclusion criteria.

**Response:**

Thank you for this comment. We excluded patients with AP due to vomiting because this condition is more consistent with acute chemical aspiration rather than dysphagia-related aspiration, which was the focus of the present study. This explanation has been added to the Methods section (Page 6, Lines 123–124).

**Comment 6:**

Additional explanation should be provided regarding the rationale for selecting the factors used as explanatory variables in the multivariate logistic regression analysis within the statistical analysis.

**Response:**

Thank you for this suggestion. We have added an explanation in the Statistical Analysis section describing the rationale for selecting explanatory variables (Page 13, Lines 282–287). Variables were chosen based on clinical relevance and temporal sequence, with baseline patient characteristics included as potential confounders and ECPS and time to SLT assessment included as process-related variables after admission. All variables were entered simultaneously using the forced-entry method.

**Comment 7:**

In the Results section, the independent presentation of “Exclusion criteria” may be confusing to readers. Presenting the number and reasons for excluded cases as part of the “Baseline patient characteristics” could help improve the overall clarity and readability of the Results.

**Response:**

Thank you for this helpful suggestion. We have integrated the information on excluded cases into the “Baseline patient characteristics” subsection of the Results (Page 14, Lines 303–305). The number of excluded patients and the final sample size are now described at the beginning of this section, with the detailed flow of patient selection presented in Figure 2.

**Comment 8:**

Page 29, line 421-423

The statement “Our patients with AP, who … in their ADLs” within the Discussion section feels abrupt. Please clarify the intent of this sentence.

**Response:**

Thank you for this comment. We have revised the sentence in the Discussion section to clarify its intent and improve the logical flow (Page 30, Lines 449–454). The revised text now explains the characteristics of the study population and the clinical context in which maintaining swallowing function may be particularly important.

**Comment 9:**

It is interesting that “neuromuscular disease” acts as an inhibitor of early oral intake in the control group. Regarding the absence of a similar effect in the ECPS group, please add a brief discussion on whether this can be interpreted as an effect of the ECPS intervention.

**Response:**

Thank you for this insightful comment. We have added a brief discussion regarding the potential interpretation of this finding (Pages 29-30, Lines 435–445). In the control group, neuromuscular disease, higher care needs, and lower FOIS scores were associated with delayed oral intake, possibly reflecting clinicians’ caution regarding aspiration risk. In contrast, in the ECPS group, decisions regarding oral intake were guided by standardized swallowing assessments and predefined criteria. This structured process may have reduced reliance on baseline clinical characteristics and instead emphasized current swallowing function.

--------------------------------

Response to Reviewer #2

--------------------------------

**Comment 1:**

Clarification of ECPS Intervention (Primary Concern)

The most important issue concerns the description of ECPS.

Although the study demonstrates a statistically significant association between ECPS implementation and early oral intake without an apparent increase in adverse outcomes, the specific procedural and structural elements of ECPS remain insufficiently described.

Terms such as “shared understanding” and “organized workflow” are abstract and do not allow readers to understand what concrete changes were implemented in daily clinical practice.

From the Introduction, it appears that SLTs were already performing early swallowing assessments prior to ECPS implementation, and that delays primarily occurred at the stage of physician decision-making. If so, ECPS may represent a standardization and streamlining of physician decision-making processes rather than an enhancement of interdisciplinary rehabilitation itself.

To improve transparency and reproducibility, please clarify:

• What specific procedural changes were introduced in physician ordering processes

• Whether predefined criteria for oral intake initiation were established

• How decision-making authority differed before and after ECPS

• What concrete elements distinguish ECPS from prior usual care

A clearer description would allow readers to understand whether ECPS represents enhanced collaboration, care pathway redesign, or decision-making standardization.

**Response:**

Thank you for this important question.

First, we address the reviewer’s comments regarding (1) the specific procedural changes introduced in physician ordering processes and (2) the concrete elements that distinguish ECPS from prior usual care. We have substantially expanded the description of the ECPS intervention in the Methods section to clarify its procedural and structural components (Pages 7–8, Lines 161–183). The revised text now specifies the interprofessional agreement, the standardized management protocol incorporated into the AP treatment protocol, and the coordinated clinical workflow, including physician ordering procedures and SLT response processes. In addition, we have clarified how ECPS differs from usual care in the control group, where decisions regarding swallowing assessment and initiation of oral intake were made at the discretion of individual physicians without a predefined protocol.

Next, we would like to address the reviewer’s comments regarding (1) whether predefined criteria for oral intake initiation were established and (2) how decision-making authority differed before and after ECPS. We have clarified the decision-making structure and the criteria used for initiating oral intake. In our hospital, under Japanese regulations, SLTs perform swallowing assessments under physician supervision, and both swallowing assessment and initiation of oral intake require a physician’s authorization (Page 4, Lines 71–73). Therefore, the formal decision-making authority did not change with ECPS implementation. In addition, eligibility for oral intake was determined according to predefined institutional criteria developed by SLTs based on standardized swallowing assessments (Page 6, Lines 136–138; Pages 8-9, Lines 184–187). These criteria remained unchanged throughout the study period. ECPS primarily reorganized and standardized the clinical workflow and timing of decision-making rather than modifying the clinical criteria or decision-making authority.

**Comment 2:**

Conceptual Framing of the Research Question

Because ECPS was explicitly designed to facilitate earlier initiation of oral intake, and early oral intake was selected as the primary outcome, the research question appears somewhat self-contained.

In other words, the intervention was structured to accelerate oral intake, and the outcome measures acceleration of oral intake. While this does not invalidate the study, clarifying that the manuscript evaluates the effectiveness of a standardized care pathway intended to accelerate decision-making—rather than demonstrating rehabilitative efficacy per se—would strengthen the conceptual framing.

Repositioning the study as an evaluation of a structured clinical pathway may enhance interpretability.

**Response:**

Thank you for this insightful comment. We agree that the conceptual framing of the study should emphasize evaluation of a structured clinical pathway rather than rehabilitative efficacy itself. We have therefore revised the Introduction and related sections of the manuscript to clarify that ECPS represents a structured interprofessional clinical pathway that is designed to standardize decision-making and to streamline the workflow from admission to initiation of oral intake. These revisions have been made in the Introduction and related sections (Page 2, Lines 27–31, 45; Page 4, Lines 83–92; Page 29, Lines 394–395; Page 33, Lines 526–527).

**Comment 3:**

Inconsistencies in Study Period Description

There are inconsistencies in the description of the study period across sections of the manuscript.

• The Study Design section describes the study as conducted between October 2019 and March 2022.

• The Exposure section refers to a period from February 2015 to February 2019.

• Data access dates are also described separately.

These discrepancies create confusion regarding the actual patient inclusion period and the timeframe used for group allocation. Because the exposure definition is directly linked to calendar time (before vs. after April 2017), precise specification of the study period is critical.

Please clarify:

• The exact patient inclusion period

• The timeframe used for exposure classification

• The relationship between data access dates and study period

Consistency across all sections is essential.

**Response:**

Thank you for pointing out this inconsistency. We have revised the Study Design and Exposure sections to clearly distinguish between the patient inclusion period and the study conduct period (Page 5, Lines 95–104). The patient inclusion period has been clarified as April 1, 2015 to March 31, 2019. This period was used for cohort identification and for exposure classification based on calendar time (before vs. after April 2017). The study conduct period (October 2019 to March 2022) refers to the timeframe during which the research project was carried out. As the data access dates are not essential to the interpretation of the study, we have removed them from the manuscript to improve clarity.

**Comment 4:**

Time-Related Confounding (Before–After Design)

The study compares patients admitted before and after April 2017. Although understandable, this design inherently introduces potential time-related confounding.

Changes in institutional policy, staffing, clinical awareness, or broader trends in pneumonia management over time may have contributed to the observed differences. This limitation should be discussed more explicitly.

Additionally, the rationale for the chosen cutoff date requires clearer justification.

**Response:**

Thank you for this important and insightful comment.

We acknowledge that the before–after design may introduce potential time-related confounding. We have therefore expanded the Limitations subsection to explicitly acknowledge this issue and to discuss the possibility that changes in institutional policies, staffing, clinical awareness, or broader trends in pneumonia management may have influenced the observed differences (Page 32, Lines 500–504).

Additionally, we have clarified the rationale for the cutoff date in the Exposure section (Page 7, Lines 153–159). April 2017 was selected because it corresponds to the time of ECPS implementation. In addition, a symmetrical observation window before and after ECPS introduction was used to partially mitigate potential secular trends over time. The study period was also limited to dates prior to the COVID-19 pandemic in Japan to avoid confounding related to pandemic-associated changes.

**Comment 5:**

Definition of Early Oral Intake

The phrase “defined as before as the initiation of oral intake within two days after admission” should be revised for clarity.

Furthermore, defining early oral intake as “within two days after admission” may lead to substantial time differences depending on admission timing (e.g., daytime vs. nighttime admission). Please clarify whether this was defined by calendar days or a fixed 48-hour window, and whether this issue was considered in the analysis.

**Response:**

Thank you for this important comment. In this retrospective study, time-of-day data for admission and initiation of oral intake were unavailable. Early oral intake was therefore operationally defined using calendar days, counting the day of admission as day 0. Initiation within two calendar days (days 0–2) was classified as early oral intake. Although this definition may allow for some imprecision compared with a strict 48-hour definition, it was applied uniformly across all participants. Thus, any potential misclassification would likely be non-differential and would tend to attenuate, rather than exaggerate, the observed association. This revision has been made in the Methods section (Page 10, Lines 219–223).

**Comment 6:**

Operational Definitions of Secondary Outcomes

Some secondary outcomes (e.g., prolonged antibiotic therapy, extended hospitalization) are not operationally defined. Please clarify how these outcomes were defined or acknowledge this as a limitation.

**Response:**

Thank you for this important comment. We did not define “prolonged antibiotic therapy” or “extended hospitalization” using specific cut-off values, as these variables were treated as continuous measures. Instead, duration of antibiotic therapy and length of hospital stay were analyzed as continuous variables and compared between groups.

To avoid potential misunderstanding, we have revised the manuscript to clarify this point and removed terminology that may imply predefined thresholds (Page 2, Line 36, 38; Page 11, Line 233, 235).

**Comment 7:**

Pre-Onset FOIS Determination

The method used to determine the pre-onset FOIS score requires further clarification. Please specify:

• The information sources used

• Who performed the assessment

• Whether a standardized mapping rule was used

This is important for reproducibility.

**Response:**

Thank you for this important comment. We have clarified the method used to determine the pre-onset FOIS score. The score was assigned retrospectively based on nursing summaries, referral letters, and admission interview records. Dietary consistency was coded according to the Japanese Dysphagia Diet 2021 established by the Japanese Society of Dysphagia Rehabilitation, and the presence of alternative nutrition was also considered using a predefined mapping rule. FOIS scoring was performed by a trained author (AT). These details have been added to the Methods section to enhance reproducibility (Pages 9–10, Lines 209–216).

**Comment 8:**

Organization of Methods

There is some overlap between the Outcome section and the “Measurements of covariates” section, and variables with different analytical roles are intermingled.

Reorganizing the Methods to clearly distinguish:

• Baseline covariates

• Primary outcome

• Secondary outcomes

• Post-admission process variables

would improve clarity and reproducibility.

**Response:**

Thank you for this helpful suggestion. We have revised the Methods section to improve clarity and to clearly distinguish variables according to their analytical roles. Specifically, the Methods section has been reorganized as you recommended. Baseline characteristics are now described separately from outcome definitions, and post-admission process variables are clearly specified. These revisions aim to improve the clarity and reproducibility of the study design. The corresponding changes have been made in the Methods section (Pages 9–11, Lines 194–250), and Tables 2 and 4 have been revised accordingly to reflect these updates.

**Comment 9:**

Sample Size and EPV

The sample size calculation assumes 10 predictor variables; however, the final multivariable model appears to include more variables.

Please clarify:

• The final number of predictors included

• The total number of outcome events

• Whether the events-per-variable criterion was satisfied based on the actual model

**Response:**

Thank you for this important comment. The final multivariable logistic regression model included 11 predictor variables. Among the 300 participants analyzed, 118 did not achieve oral intake within two days and were considered outcome events for the logistic regression analysis. Therefore, the events-per-variable (EPV) was calculated as 118/11 = 10.7, which exceeds the commonly recommended minimum threshold of 10. We have clarified this in the Results section (page 26, lines 378–381).

**Minor Comments**

• Improve the clarity and resolution of Figures so that the workflow differences between groups are more clearly illustrated.

• Revise minor grammatical issues for clarity.

**Response:**

Thank you for your suggestions. We have addressed the Figures as requested, and we have worked closely with a professional editor to tighten up the text.

We believe that these revisions have substantially improved the clarity and robustness of the manuscript.
