# Supplementary material for: Enhanced collaboration of physicians and speech-language therapists promotes early oral intake in older adults with aspiration pneumonia: A retrospective cohort study
Source: PLoS One. 2026 Jun 10;21(6):e0350495. doi: 10.1371/journal.pone.0350495 (PMC13252771; doi:10.1371/journal.pone.0350495)
Supplement: Attachment — Submitted filename: Response_to_Reviewers_auresp_2.docx [file pone.0350495.s005.docx]

Dear Editor,

We sincerely thank you and the reviewers for your positive and constructive comments and suggestions, and we are pleased to learn that our most recent draft has been received favorably.

In response to your further comments and suggestions, we have carefully revised the manuscript again. Our detailed responses are provided below.

--------------------------------

Response to Reviewer #1

--------------------------------

**Comment:**

Thank you for your thorough and appropriate responses to our comments. The revised manuscript has been substantially improved, and most of the concerns raised in the initial review have been adequately addressed.

Therefore, I believe that the manuscript is now suitable for publication in its current form.

One minor point for your consideration: in the revised submission, changes were indicated using the Word “Track Changes” function rather than by clearly marking revisions (e.g., in red text), which is more commonly used. While this approach is acceptable, it can be somewhat difficult for reviewers to follow the revisions. I would recommend adopting a clearer method for highlighting changes in future submissions.

**Response:**

We thank the reviewer for the positive and encouraging comments. We have taken on board your suggestion of marking the changes in red rather than using the track changes function. We will consider this in future submissions to make the review process more convenient for the reviewers.

--------------------------------

Response to Reviewer #2

--------------------------------

**Comment:**

Thank you for the detailed revision. The clarified description of the ECPS workflow has improved my understanding, and the distinction from usual care is now much clearer.

In particular, it appears that a key feature of this intervention is the alignment of physician orders for both SLT assessment and diet initiation at an early stage, which enables timely initiation of oral intake.

I would be inclined to accept this explanation. However, as the authors describe ECPS as “enhanced collaboration,” it would be helpful to further clarify how the inter-professional agreement was established in practice. Specifically, some additional information on how this shared approach was initiated and implemented within the institution would improve transparency and may enhance the reproducibility of the intervention.

If possible, a brief description of this process would be valuable.

**Response:**

Thank you for this insightful comment. In response, we have added a more detailed description of how the inter-professional agreement underlying ECPS was established and implemented in practice.

Specifically, we have clarified that the agreement was developed through interdisciplinary discussions and consensus among physicians and speech-language therapists within the institution, based on a shared recognition informed by previous studies. We have also clarified that this agreement was directly incorporated into the institutional AP treatment protocol and implemented in routine clinical practice without delay (Page 8, Lines 163–171).

We believe that these revisions have substantially improved the clarity and robustness of the manuscript and we are looking forward to hearing from you again soon.
