# Supplementary material for: Enhanced collaboration of physicians and speech-language therapists promotes early oral intake in older adults with aspiration pneumonia: A retrospective cohort study
Source: PLoS One. 2026 Jun 10;21(6):e0350495. doi: 10.1371/journal.pone.0350495 (PMC13252771; doi:10.1371/journal.pone.0350495)
Supplement: Peer review history

# Peer review: Decision Letter 0

**DOI:** 10.1371/journal.pone.0350495.r001

**Authors:** Jennifer Tucker

16 Feb 2026

PONE-D-25-64667

Enhanced collaboration of physicians and speech-language therapists promotes early oral intake in older adults with aspiration pneumonia: a retrospective cohort study

PLOS One

Dear Dr. Tawaraya,

Thank you for submitting your manuscript to PLOS ONE. After careful consideration, we feel that it has merit but does not fully meet PLOS ONE’s publication criteria as it currently stands. Therefore, we invite you to submit a revised version of the manuscript that addresses the points raised during the review process.

Please submit your revised manuscript by Apr 02 2026 11:59PM. If you will need more time than this to complete your revisions, please reply to this message or contact the journal office at plosone@plos.org. When you're ready to submit your revision, log on to https://www.editorialmanager.com/pone/ and select the 'Submissions Needing Revision' folder to locate your manuscript file.

Please include the following items when submitting your revised manuscript:

- A letter that responds to each point raised by the academic editor and reviewer(s). You should upload this letter as a separate file labeled 'Response to Reviewers'.
- A marked-up copy of your manuscript that highlights changes made to the original version. You should upload this as a separate file labeled 'Revised Manuscript with Track Changes'.
- An unmarked version of your revised paper without tracked changes. You should upload this as a separate file labeled 'Manuscript'.

If you would like to make changes to your financial disclosure, please include your updated statement in your cover letter. Guidelines for resubmitting your figure files are available below the reviewer comments at the end of this letter.

If applicable, we recommend that you deposit your laboratory protocols in protocols.io to enhance the reproducibility of your results. Protocols.io assigns your protocol its own identifier (DOI) so that it can be cited independently in the future. For instructions see: https://journals.plos.org/plosone/s/submission-guidelines#loc-laboratory-protocols. Additionally, PLOS ONE offers an option for publishing peer-reviewed Lab Protocol articles, which describe protocols hosted on protocols.io. Read more information on sharing protocols at https://plos.org/protocols?utm_medium=editorial-email&utm_source=authorletters&utm_campaign=protocols.

We look forward to receiving your revised manuscript.

Kind regards,

Jennifer Tucker, PhD

Staff Editor

PLOS One

Journal Requirements:

When submitting your revision, we need you to address these additional requirements.

1.Please ensure that your manuscript meets PLOS ONE's style requirements, including those for file naming. The PLOS ONE style templates can be found at

https://journals.plos.org/plosone/s/file?id=wjVg/PLOSOne_formatting_sample_main_body.pdf and

https://journals.plos.org/plosone/s/file?id=ba62/PLOSOne_formatting_sample_title_authors_affiliations.pdf

2. We note that your Data Availability Statement is currently as follows: All relevant data are within the manuscript and in Supporting Information files.

Please confirm at this time whether or not your submission contains all raw data required to replicate the results of your study. Authors must share the “minimal data set” for their submission. PLOS defines the minimal data set to consist of the data required to replicate all study findings reported in the article, as well as related metadata and methods (https://journals.plos.org/plosone/s/data-availability#loc-minimal-data-set-definition).

For example, authors should submit the following data:

- The values behind the means, standard deviations and other measures reported;

- The values used to build graphs;

- The points extracted from images for analysis.

Authors do not need to submit their entire data set if only a portion of the data was used in the reported study.

If your submission does not contain these data, please either upload them as Supporting Information files or deposit them to a stable, public repository and provide us with the relevant URLs, DOIs, or accession numbers. For a list of recommended repositories, please see https://journals.plos.org/plosone/s/recommended-repositories.

If there are ethical or legal restrictions on sharing a de-identified data set, please explain them in detail (e.g., data contain potentially sensitive information, data are owned by a third-party organization, etc.) and who has imposed them (e.g., an ethics committee). Please also provide contact information for a data access committee, ethics committee, or other institutional body to which data requests may be sent. If data are owned by a third party, please indicate how others may request data access.

3. When completing the data availability statement of the submission form, you indicated that you will make your data available on acceptance. We strongly recommend all authors decide on a data sharing plan before acceptance, as the process can be lengthy and hold up publication timelines. Please note that, though access restrictions are acceptable now, your entire data will need to be made freely accessible if your manuscript is accepted for publication. This policy applies to all data except where public deposition would breach compliance with the protocol approved by your research ethics board. If you are unable to adhere to our open data policy, please kindly revise your statement to explain your reasoning and we will seek the editor's input on an exemption. Please be assured that, once you have provided your new statement, the assessment of your exemption will not hold up the peer review process.

4. Please amend the manuscript submission data (via Edit Submission) to include author Atsushi Keyaki.

5. If the reviewer comments include a recommendation to cite specific previously published works, please review and evaluate these publications to determine whether they are relevant and should be cited. There is no requirement to cite these works unless the editor has indicated otherwise.

Additional Editor Comments:

-->The manuscript has been evaluated by two reviewers, and their comments are available below.

Could you please carefully revise the manuscript to address all comments raised?

[Note: HTML markup is below. Please do not edit.]

Reviewers' comments:

Reviewer's Responses to Questions

**Comments to the Author**

1. Is the manuscript technically sound, and do the data support the conclusions?

The manuscript must describe a technically sound piece of scientific research with data that supports the conclusions. Experiments must have been conducted rigorously, with appropriate controls, replication, and sample sizes. The conclusions must be drawn appropriately based on the data presented.

Reviewer #1: Yes

Reviewer #2: Yes

**********

2. Has the statistical analysis been performed appropriately and rigorously?

Reviewer #1: Yes

Reviewer #2: Yes

**********

3. Have the authors made all data underlying the findings in their manuscript fully available?

The PLOS Data policy requires authors to make all data underlying the findings described in their manuscript fully available without restriction, with rare exception (please refer to the Data Availability Statement in the manuscript PDF file). The data should be provided as part of the manuscript or its supporting information, or deposited to a public repository. For example, in addition to summary statistics, the data points behind means, medians and variance measures should be available. If there are restrictions on publicly sharing data—e.g. participant privacy or use of data from a third party—those must be specified.

Reviewer #1: Yes

Reviewer #2: Yes

**********

4. Is the manuscript presented in an intelligible fashion and written in standard English?

PLOS ONE does not copyedit accepted manuscripts, so the language in submitted articles must be clear, correct, and unambiguous. Any typographical or grammatical errors should be corrected at revision, so please note any specific errors here.

Reviewer #1: Yes

Reviewer #2: Yes

**********

5. Review Comments to the Author

Please use the space provided to explain your answers to the questions above. You may also include additional comments for the author, including concerns about dual publication, research ethics, or publication ethics. (Please upload your review as an attachment if it exceeds 20,000 characters)

Reviewer #1: This study presents the interesting finding that active intervention by speech-language therapists (SLTs) in patients with aspiration pneumonia may facilitate early oral intake and be associated with subsequent patient outcomes. Against the backdrop of increasing aspiration pneumonia patients in Japan, this study addresses an important theme. However, the writing is generally redundant and requires appropriate revisions, including improvements to its structure.

Major comments

1. The fundamental differences between the ECPS group and the control group are unclear. The decision-making process within the ECPS group should be described more specifically. Is it correct to understand that the difference between the two groups lies solely in who decides the timing of initiating oral intake? Were diet orders for both groups placed by physicians? The meaning of “a diet order was placed simultaneously with the referral to the SLT (Page 8, line 184-185)” is unclear. Additionally, clarification is needed on whether there were substantive differences between the groups in subsequent rehabilitation content or interventions.

However, the paragraph on “Dysphagia rehabilitation system at our hospital” is somewhat redundant; it would be preferable to reorganize and summarize it for clarity.

2. SLT initiation generally occurred within 1 (1-2) days in both the ECPS and control groups. A statistically significant difference existed between the groups, and the authors demonstrated, based on multivariate analysis, that delayed SLT initiation delayed oral intake initiation by 31%. However, questions remain regarding the clinical significance of this “difference within one day (or a matter of hours).” For example, in sepsis, the importance of initiating antimicrobial therapy within one hour of diagnosis is debated. Similarly, in this study, it requires consideration of whether the intervention at the time unit level demonstrates clinical utility.

3. In the ECPS group and the early oral intake group, reductions were observed in “resumption of antibiotic therapy,” “length of hospital stay,” and “duration of antimicrobial therapy.” While it is unlikely that SLT directly influenced the duration of antimicrobial administration, consideration is warranted regarding the potential impact of early initiation of oral intake on these outcomes.

4. The entire section in Discussion is redundant, with the second ”Multivariate analysis…” and third “Our exploratory analysis…” paragraphs largely consisting of restating the results. It would be preferable to concisely summarize these points and then logically transition to the discussion in the fourth paragraph “From a swallowing…”.

5. While the importance of collaboration with SLTs and physicians is understood, it is anticipated that some medical institutions may be unable to establish similar systems due to manpower or facility constraints. Based on the results of this study, please briefly discuss the patient backgrounds and clinical factors that could serve as reference points when considering initiating oral intake outside of ECPS.

Minor comments

1. The “Study design and setting” section lists the study period and data access period, while the ‘Exposure’ section indicates the patient enrollment period. However, these descriptions are scattered and difficult to understand. It is desirable to clarify why the study period and data access period differ, and to consolidate the description including the patient enrollment period into the “Study design and setting” section.

2. Page 6, line 120

“…and upper respiratory tract symptoms such as sputum production”

Sputum production may correspond to “lower” respiratory tract symptoms.

3. Page 9, line 189-191

“In contrast, patients in the control group… and the timing of swallowing assessment and oral intake was determined at the discretion of the attending physician”

The statement in the introduction that “Even before the implementation of this protocol, SLTs at our hospital routinely conducted swallowing assessment at an early stage” appears contradictory. The wording needs to be revised.

4. Page 9, line 200

“…defined as before as the initiation of oral intake within two days after admission”

“as before” seems grammatically inappropriate.

5. While episodes of aspiration are useful evidence for suspecting aspiration pneumonia, please clarify the reason for excluding “patients with AP due to vomiting” in the exclusion criteria.

6. Additional explanation should be provided regarding the rationale for selecting the factors used as explanatory variables in the multivariate logistic regression analysis within the statistical analysis.

7. In the Results section, the independent presentation of “Exclusion criteria” may be confusing to readers. Presenting the number and reasons for excluded cases as part of the “Baseline patient characteristics” could help improve the overall clarity and readability of the Results.

8. Page 29, line 421-423

The statement “Our patients with AP, who … in their ADLs” within the Discussion section feels abrupt. Please clarify the intent of this sentence.

9. It is interesting that “neuromuscular disease” acts as an inhibitor of early oral intake in the control group. Regarding the absence of a similar effect in the ECPS group, please add a brief discussion on whether this can be interpreted as an effect of the ECPS intervention.

Reviewer #2: Overall assessment

This study evaluates the association between the implementation of enhanced collaboration between physicians and speech-language therapists (ECPS) and early initiation of oral intake in older adults with aspiration pneumonia. The dataset is substantial, and the statistical analyses are generally appropriate. The findings are clinically interesting. With clarification and refinement in several key areas—particularly regarding the definition and positioning of ECPS—the manuscript has the potential to become a valuable contribution.

________________________________________

Major Comments

1. Clarification of ECPS Intervention (Primary Concern)

The most important issue concerns the description of ECPS.

Although the study demonstrates a statistically significant association between ECPS implementation and early oral intake without an apparent increase in adverse outcomes, the specific procedural and structural elements of ECPS remain insufficiently described.

Terms such as “shared understanding” and “organized workflow” are abstract and do not allow readers to understand what concrete changes were implemented in daily clinical practice.

From the Introduction, it appears that SLTs were already performing early swallowing assessments prior to ECPS implementation, and that delays primarily occurred at the stage of physician decision-making. If so, ECPS may represent a standardization and streamlining of physician decision-making processes rather than an enhancement of interdisciplinary rehabilitation itself.

To improve transparency and reproducibility, please clarify:

• What specific procedural changes were introduced in physician ordering processes

• Whether predefined criteria for oral intake initiation were established

• How decision-making authority differed before and after ECPS

• What concrete elements distinguish ECPS from prior usual care

A clearer description would allow readers to understand whether ECPS represents enhanced collaboration, care pathway redesign, or decision-making standardization.

________________________________________

2. Conceptual Framing of the Research Question

Because ECPS was explicitly designed to facilitate earlier initiation of oral intake, and early oral intake was selected as the primary outcome, the research question appears somewhat self-contained.

In other words, the intervention was structured to accelerate oral intake, and the outcome measures acceleration of oral intake. While this does not invalidate the study, clarifying that the manuscript evaluates the effectiveness of a standardized care pathway intended to accelerate decision-making—rather than demonstrating rehabilitative efficacy per se—would strengthen the conceptual framing.

Repositioning the study as an evaluation of a structured clinical pathway may enhance interpretability.

________________________________________

3. Inconsistencies in Study Period Description

There are inconsistencies in the description of the study period across sections of the manuscript.

• The Study Design section describes the study as conducted between October 2019 and March 2022.

• The Exposure section refers to a period from February 2015 to February 2019.

• Data access dates are also described separately.

These discrepancies create confusion regarding the actual patient inclusion period and the timeframe used for group allocation. Because the exposure definition is directly linked to calendar time (before vs. after April 2017), precise specification of the study period is critical.

Please clarify:

• The exact patient inclusion period

• The timeframe used for exposure classification

• The relationship between data access dates and study period

Consistency across all sections is essential.

________________________________________

4. Time-Related Confounding (Before–After Design)

The study compares patients admitted before and after April 2017. Although understandable, this design inherently introduces potential time-related confounding.

Changes in institutional policy, staffing, clinical awareness, or broader trends in pneumonia management over time may have contributed to the observed differences. This limitation should be discussed more explicitly.

Additionally, the rationale for the chosen cutoff date requires clearer justification.

________________________________________

5. Definition of Early Oral Intake

The phrase “defined as before as the initiation of oral intake within two days after admission” should be revised for clarity.

Furthermore, defining early oral intake as “within two days after admission” may lead to substantial time differences depending on admission timing (e.g., daytime vs. nighttime admission). Please clarify whether this was defined by calendar days or a fixed 48-hour window, and whether this issue was considered in the analysis.

________________________________________

6. Operational Definitions of Secondary Outcomes

Some secondary outcomes (e.g., prolonged antibiotic therapy, extended hospitalization) are not operationally defined. Please clarify how these outcomes were defined or acknowledge this as a limitation.

________________________________________

7. Pre-Onset FOIS Determination

The method used to determine the pre-onset FOIS score requires further clarification. Please specify:

• The information sources used

• Who performed the assessment

• Whether a standardized mapping rule was used

This is important for reproducibility.

________________________________________

8. Organization of Methods

There is some overlap between the Outcome section and the “Measurements of covariates” section, and variables with different analytical roles are intermingled.

Reorganizing the Methods to clearly distinguish:

• Baseline covariates

• Primary outcome

• Secondary outcomes

• Post-admission process variables

would improve clarity and reproducibility.

________________________________________

9. Sample Size and EPV

The sample size calculation assumes 10 predictor variables; however, the final multivariable model appears to include more variables.

Please clarify:

• The final number of predictors included

• The total number of outcome events

• Whether the events-per-variable criterion was satisfied based on the actual model

________________________________________

Minor Comments

• Improve the clarity and resolution of Figures so that the workflow differences between groups are more clearly illustrated.

• Revise minor grammatical issues for clarity.

________________________________________

Final Recommendation

This is a well-conducted retrospective analysis with clinically relevant findings. The primary area requiring improvement is the clearer specification and positioning of the ECPS intervention, along with clarification of study period inconsistencies. Addressing these issues would substantially strengthen the manuscript.

**********

6. PLOS authors have the option to publish the peer review history of their article (what does this mean?). If published, this will include your full peer review and any attached files.

If you choose “no”, your identity will remain anonymous but your review may still be made public.

**Do you want your identity to be public for this peer review?** For information about this choice, including consent withdrawal, please see our Privacy Policy.

Reviewer #1: No

Reviewer #2: No

**********

[NOTE: If reviewer comments were submitted as an attachment file, they will be attached to this email and accessible via the submission site. Please log into your account, locate the manuscript record, and check for the action link "View Attachments". If this link does not appear, there are no attachment files.]

To ensure your figures meet our technical requirements, please review our figure guidelines: https://journals.plos.org/plosone/s/figures

You may also use PLOS’s free figure tool, NAAS, to help you prepare publication quality figures: https://journals.plos.org/plosone/s/figures#loc-tools-for-figure-preparation.

NAAS will assess whether your figures meet our technical requirements by comparing each figure against our figure specifications.

Attachment Submitted filename: renamed_f9540.docx

Attachment Submitted filename: Review tazawa.docx

# Author comment: Author response to Decision Letter 1

**DOI:** 10.1371/journal.pone.0350495.r002

30 Mar 2026

We have provided a detailed point-by-point response to all reviewer and editor comments in the uploaded response-to-reviewers document.

Attachment Submitted filename: Response to Reviewers.docx

# Peer review: Decision Letter 1

**DOI:** 10.1371/journal.pone.0350495.r003

**Authors:** Ilse Bloom

15 Apr 2026

PONE-D-25-64667R1

Enhanced collaboration of physicians and speech-language therapists promotes early oral intake in older adults with aspiration pneumonia: a retrospective cohort study

PLOS One

Dear Dr. Tawaraya,

Thank you for submitting your manuscript to PLOS ONE. After careful consideration, we feel that it has merit but does not fully meet PLOS ONE’s publication criteria as it currently stands. Therefore, we invite you to submit a revised version of the manuscript that addresses the points raised during the review process.

The manuscript has been further evaluated by two reviewers, and their comments are available below.

Could you please carefully revise the manuscript to address all comments raised?

Please submit your revised manuscript by May 29 2026 11:59PM. If you will need more time than this to complete your revisions, please reply to this message or contact the journal office at plosone@plos.org. When you're ready to submit your revision, log on to https://www.editorialmanager.com/pone/ and select the 'Submissions Needing Revision' folder to locate your manuscript file.

Please include the following items when submitting your revised manuscript:

- A letter that responds to each point raised by the academic editor and reviewer(s). You should upload this letter as a separate file labeled 'Response to Reviewers'.
- A marked-up copy of your manuscript that highlights changes made to the original version. You should upload this as a separate file labeled 'Revised Manuscript with Track Changes'.
- An unmarked version of your revised paper without tracked changes. You should upload this as a separate file labeled 'Manuscript'.

If you would like to make changes to your financial disclosure, please include your updated statement in your cover letter. Guidelines for resubmitting your figure files are available below the reviewer comments at the end of this letter.

If applicable, we recommend that you deposit your laboratory protocols in protocols.io to enhance the reproducibility of your results. Protocols.io assigns your protocol its own identifier (DOI) so that it can be cited independently in the future. For instructions see: https://journals.plos.org/plosone/s/submission-guidelines#loc-laboratory-protocols. Additionally, PLOS ONE offers an option for publishing peer-reviewed Lab Protocol articles, which describe protocols hosted on protocols.io. Read more information on sharing protocols at https://plos.org/protocols?utm_medium=editorial-email&utm_source=authorletters&utm_campaign=protocols.

As the corresponding author, your ORCID iD is verified in the submission system and will appear in the published article. PLOS supports the use of ORCID, and we encourage all coauthors to register for an ORCID iD and use it as well. Please encourage your coauthors to verify their ORCID iD within the submission system before final acceptance, as unverified ORCID iDs will not appear in the published article. *Only* the individual author can complete the verification step; PLOS staff *cannot* verify ORCID iDs on behalf of authors.

We look forward to receiving your revised manuscript.

Kind regards,

Ilse Bloom

Staff Editor

PLOS One

Journal Requirements:

1. If the reviewer comments include a recommendation to cite specific previously published works, please review and evaluate these publications to determine whether they are relevant and should be cited. There is no requirement to cite these works unless the editor has indicated otherwise.

2. Please review your reference list to ensure that it is complete and correct. If you have cited papers that have been retracted, please include the rationale for doing so in the manuscript text, or remove these references and replace them with relevant current references. Any changes to the reference list should be mentioned in the rebuttal letter that accompanies your revised manuscript. If you need to cite a retracted article, indicate the article’s retracted status in the References list and also include a citation and full reference for the retraction notice.

[Note: HTML markup is below. Please do not edit.]

Reviewers' comments:

Reviewer's Responses to Questions

**Comments to the Author**

1. If the authors have adequately addressed your comments raised in a previous round of review and you feel that this manuscript is now acceptable for publication, you may indicate that here to bypass the “Comments to the Author” section, enter your conflict of interest statement in the “Confidential to Editor” section, and submit your "Accept" recommendation.

Reviewer #1: All comments have been addressed

Reviewer #2: (No Response)

**********

2. Is the manuscript technically sound, and do the data support the conclusions?

The manuscript must describe a technically sound piece of scientific research with data that supports the conclusions. Experiments must have been conducted rigorously, with appropriate controls, replication, and sample sizes. The conclusions must be drawn appropriately based on the data presented.

Reviewer #1: Yes

Reviewer #2: Yes

**********

3. Has the statistical analysis been performed appropriately and rigorously?

Reviewer #1: Yes

Reviewer #2: Yes

**********

4. Have the authors made all data underlying the findings in their manuscript fully available?

The PLOS Data policy requires authors to make all data underlying the findings described in their manuscript fully available without restriction, with rare exception (please refer to the Data Availability Statement in the manuscript PDF file). The data should be provided as part of the manuscript or its supporting information, or deposited to a public repository. For example, in addition to summary statistics, the data points behind means, medians and variance measures should be available. If there are restrictions on publicly sharing data—e.g. participant privacy or use of data from a third party—those must be specified.

Reviewer #1: Yes

Reviewer #2: Yes

**********

5. Is the manuscript presented in an intelligible fashion and written in standard English?

PLOS ONE does not copyedit accepted manuscripts, so the language in submitted articles must be clear, correct, and unambiguous. Any typographical or grammatical errors should be corrected at revision, so please note any specific errors here.

Reviewer #1: Yes

Reviewer #2: Yes

**********

6. Review Comments to the Author

Please use the space provided to explain your answers to the questions above. You may also include additional comments for the author, including concerns about dual publication, research ethics, or publication ethics. (Please upload your review as an attachment if it exceeds 20,000 characters)

Reviewer #1: Thank you for your thorough and appropriate responses to our comments. The revised manuscript has been substantially improved, and most of the concerns raised in the initial review have been adequately addressed.

Therefore, I believe that the manuscript is now suitable for publication in its current form.

One minor point for your consideration: in the revised submission, changes were indicated using the Word “Track Changes” function rather than by clearly marking revisions (e.g., in red text), which is more commonly used. While this approach is acceptable, it can be somewhat difficult for reviewers to follow the revisions. I would recommend adopting a clearer method for highlighting changes in future submissions.

Reviewer #2: Comment 1:

Thank you for the detailed revision. The clarified description of the ECPS workflow has improved my understanding, and the distinction from usual care is now much clearer.

In particular, it appears that a key feature of this intervention is the alignment of physician orders for both SLT assessment and diet initiation at an early stage, which enables timely initiation of oral intake.

I would be inclined to accept this explanation. However, as the authors describe ECPS as “enhanced collaboration,” it would be helpful to further clarify how the inter-professional agreement was established in practice. Specifically, some additional information on how this shared approach was initiated and implemented within the institution would improve transparency and may enhance the reproducibility of the intervention.

If possible, a brief description of this process would be valuable.

**********

7. PLOS authors have the option to publish the peer review history of their article (what does this mean?). If published, this will include your full peer review and any attached files.

If you choose “no”, your identity will remain anonymous but your review may still be made public.

**Do you want your identity to be public for this peer review?** For information about this choice, including consent withdrawal, please see our Privacy Policy.

Reviewer #1: No

Reviewer #2: No

**********

[NOTE: If reviewer comments were submitted as an attachment file, they will be attached to this email and accessible via the submission site. Please log into your account, locate the manuscript record, and check for the action link "View Attachments". If this link does not appear, there are no attachment files.]

To ensure your figures meet our technical requirements, please review our figure guidelines: https://journals.plos.org/plosone/s/figures

You may also use PLOS’s free figure tool, NAAS, to help you prepare publication quality figures: https://journals.plos.org/plosone/s/figures#loc-tools-for-figure-preparation.

NAAS will assess whether your figures meet our technical requirements by comparing each figure against our figure specifications.

# Author comment: Author response to Decision Letter 2

**DOI:** 10.1371/journal.pone.0350495.r004

28 Apr 2026

--------------------------------

Response to Reviewer #1

--------------------------------

Comment:

Thank you for your thorough and appropriate responses to our comments. The revised manuscript has been substantially improved, and most of the concerns raised in the initial review have been adequately addressed.

Therefore, I believe that the manuscript is now suitable for publication in its current form.

One minor point for your consideration: in the revised submission, changes were indicated using the Word “Track Changes” function rather than by clearly marking revisions (e.g., in red text), which is more commonly used. While this approach is acceptable, it can be somewhat difficult for reviewers to follow the revisions. I would recommend adopting a clearer method for highlighting changes in future submissions.

Response:

We thank the reviewer for the positive and encouraging comments. We have taken on board your suggestion of marking the changes in red rather than using the track changes function. We will consider this in future submissions to make the review process more convenient for the reviewers.

--------------------------------

Response to Reviewer #2

--------------------------------

Comment:

Thank you for the detailed revision. The clarified description of the ECPS workflow has improved my understanding, and the distinction from usual care is now much clearer.

In particular, it appears that a key feature of this intervention is the alignment of physician orders for both SLT assessment and diet initiation at an early stage, which enables timely initiation of oral intake.

I would be inclined to accept this explanation. However, as the authors describe ECPS as “enhanced collaboration,” it would be helpful to further clarify how the inter-professional agreement was established in practice. Specifically, some additional information on how this shared approach was initiated and implemented within the institution would improve transparency and may enhance the reproducibility of the intervention.

If possible, a brief description of this process would be valuable.

Response:

Thank you for this insightful comment. In response, we have added a more detailed description of how the inter-professional agreement underlying ECPS was established and implemented in practice.

Specifically, we have clarified that the agreement was developed through interdisciplinary discussions and consensus among physicians and speech-language therapists within the institution, based on a shared recognition informed by previous studies. We have also clarified that this agreement was directly incorporated into the institutional AP treatment protocol and implemented in routine clinical practice without delay (Page 8, Lines 163–171).

Attachment Submitted filename: Response_to_Reviewers_auresp_2.docx

# Peer review: Decision Letter 2

**DOI:** 10.1371/journal.pone.0350495.r005

**Authors:** Marianne Clemence

15 May 2026

Enhanced collaboration of physicians and speech-language therapists promotes early oral intake in older adults with aspiration pneumonia: a retrospective cohort study

PONE-D-25-64667R2

Dear Dr. Tawaraya,

We’re pleased to inform you that your manuscript has been judged scientifically suitable for publication and will be formally accepted for publication once it meets all outstanding technical requirements.

Within one week, you’ll receive an e-mail detailing the required amendments. When these have been addressed, you’ll receive a formal acceptance letter and your manuscript will be scheduled for publication.

An invoice will be generated when your article is formally accepted. Please note, if your institution has a publishing partnership with PLOS and your article meets the relevant criteria, all or part of your publication costs will be covered. Please make sure your user information is up-to-date by logging into Editorial Manager at Editorial Manager® and clicking the ‘Update My Information' link at the top of the page. For questions related to billing, please contact billing support.

If your institution or institutions have a press office, please notify them about your upcoming paper to help maximize its impact. If they’ll be preparing press materials, please inform our press team as soon as possible -- no later than 48 hours after receiving the formal acceptance. Your manuscript will remain under strict press embargo until 2 pm Eastern Time on the date of publication. For more information, please contact onepress@plos.org.

Kind regards,

Marianne Clemence

Staff Editor

PLOS One

Additional Editor Comments (optional):

Reviewers' comments:

Reviewer's Responses to Questions

**Comments to the Author**

1. If the authors have adequately addressed your comments raised in a previous round of review and you feel that this manuscript is now acceptable for publication, you may indicate that here to bypass the “Comments to the Author” section, enter your conflict of interest statement in the “Confidential to Editor” section, and submit your "Accept" recommendation.

Reviewer #1: All comments have been addressed

Reviewer #2: All comments have been addressed

**********

2. Is the manuscript technically sound, and do the data support the conclusions?

The manuscript must describe a technically sound piece of scientific research with data that supports the conclusions. Experiments must have been conducted rigorously, with appropriate controls, replication, and sample sizes. The conclusions must be drawn appropriately based on the data presented.

Reviewer #1: Yes

Reviewer #2: Yes

**********

3. Has the statistical analysis been performed appropriately and rigorously?

Reviewer #1: Yes

Reviewer #2: Yes

**********

4. Have the authors made all data underlying the findings in their manuscript fully available?

The PLOS Data policy requires authors to make all data underlying the findings described in their manuscript fully available without restriction, with rare exception (please refer to the Data Availability Statement in the manuscript PDF file). The data should be provided as part of the manuscript or its supporting information, or deposited to a public repository. For example, in addition to summary statistics, the data points behind means, medians and variance measures should be available. If there are restrictions on publicly sharing data—e.g. participant privacy or use of data from a third party—those must be specified.

Reviewer #1: Yes

Reviewer #2: Yes

**********

5. Is the manuscript presented in an intelligible fashion and written in standard English?

PLOS ONE does not copyedit accepted manuscripts, so the language in submitted articles must be clear, correct, and unambiguous. Any typographical or grammatical errors should be corrected at revision, so please note any specific errors here.

Reviewer #1: Yes

Reviewer #2: Yes

**********

6. Review Comments to the Author

Please use the space provided to explain your answers to the questions above. You may also include additional comments for the author, including concerns about dual publication, research ethics, or publication ethics. (Please upload your review as an attachment if it exceeds 20,000 characters)

Reviewer #1: Thank you for the opportunity to review the revised manuscript. I have no further comments, and I have no concerns regarding the current version.

Reviewer #2: Thank you for the opportunity to review this manuscript. The study addresses an important and clinically relevant question regarding early oral intake in older adults with aspiration pneumonia.

I appreciate the authors’ careful and thoughtful revisions in response to the previous comments. In particular, the additional description of the inter-professional agreement underlying ECPS has improved the transparency of the manuscript and clarified how the approach was implemented in practice.

While the description of the agreement process remains somewhat general, I believe the current level of detail is sufficient for understanding the intervention in the context of this study.

Overall, the manuscript has been substantially improved through revision, and I consider it suitable for publication in its current form.

**********

7. PLOS authors have the option to publish the peer review history of their article (what does this mean?). If published, this will include your full peer review and any attached files.

If you choose “no”, your identity will remain anonymous but your review may still be made public.

**Do you want your identity to be public for this peer review?** For information about this choice, including consent withdrawal, please see our Privacy Policy.

Reviewer #1: No

Reviewer #2: No

**********

# Editor report: Acceptance letter

**DOI:** 10.1371/journal.pone.0350495.r006

**Authors:** Marianne Clemence

PONE-D-25-64667R2

PLOS One

Dear Dr. Tawaraya,

I'm pleased to inform you that your manuscript has been deemed suitable for publication in PLOS One. Congratulations! Your manuscript is now being handed over to our production team.

At this stage, our production department will prepare your paper for publication. This includes ensuring the following:

* All references, tables, and figures are properly cited

* All relevant supporting information is included in the manuscript submission,

* There are no issues that prevent the paper from being properly typeset

You will receive further instructions from the production team, including instructions on how to review your proof when it is ready. Please keep in mind that we are working through a large volume of accepted articles, so please give us a few days to review your paper and let you know the next and final steps.

Lastly, if your institution or institutions have a press office, please let them know about your upcoming paper now to help maximize its impact. If they'll be preparing press materials, please inform our press team within the next 48 hours. Your manuscript will remain under strict press embargo until 2 pm Eastern Time on the date of publication. For more information, please contact onepress@plos.org.

You will receive an invoice from PLOS for your publication fee after your manuscript has reached the completed accept phase. If you receive an email requesting payment before acceptance or for any other service, this may be a phishing scheme. Learn how to identify phishing emails and protect your accounts at https://explore.plos.org/phishing.

If we can help with anything else, please email us at customercare@plos.org.

Thank you for submitting your work to PLOS ONE and supporting open access.

Kind regards,

PLOS ONE Editorial Office Staff

on behalf of

Dr Marianne Clemence

Staff Editor

PLOS One
